# Sex differences in the effects of calcitonin gene-related peptide signaling on migraine-like behavior in animal models: a narrative review

**DOI:** 10.3389/fneur.2025.1603758

**Published:** 2025-07-10

**Authors:** Lakeisha A. Lewter, Rachel L. Arnold, Nina B. Narosov, Gregory Dussor, Benedict J. Kolber

**Affiliations:** Department of Neuroscience, School of Behavioral and Brain Sciences, Center for Advanced Pain Studies, The University of Texas at Dallas, Dallas, TX, United States

**Keywords:** CGRP, antagonists, sex differences, headache, migraine, rodent models

## Abstract

Migraine is a common neurological disorder with a typical onset in adolescence or early adulthood. Migraine is three times more common in women than men, but the definitive cause underlying the observed disparity is not fully understood. Calcitonin gene-related peptide (CGRP) is a neuropeptide and potent vasodilator that is now clearly linked to migraine based on the efficacy of drugs targeting its signaling. While the efficacy and safety of drugs targeting CGRP are now well established, there is a shortage of studies exploring sex differences between CGRP and CGRP-based therapy related to migraine. This review evaluates the preclinical literature focusing on the effect of CGRP and inhibition of CGRP signaling on migraine-like behavior in male and female rodents. For this review, PubMed database was searched using the following terms: “CGRP AND Migraine AND animal models.” Papers were selected for review and risk of bias (RoB) assessment to evaluate the central question – What sex differences in CGRP signaling and migraine-like behavior are observed in rodents? CGRP itself induces pronociceptive effects in both male and female mice but when considering studies that directly compared male and females, there is a case for stronger overall effects in female rodents. Inhibition of CGRP signaling has a primarily antinociceptive effect in studies using only male or female rodents. We highlight that very few studies are conducted with adequate statistical power to measure sex differences within a single study and several studies pool mice across sexes. Given the known sex differences in the human condition, this pooling methodology may not be best practice for future studies involved CGRP in rodents. Overall, while there is clinical evidence suggesting therapeutics targeting CGRP could possibly have different gendered effects in humans, more preclinical studies need to be conducted to understand sex differences in CGRP or CGRP antagonism in migraine-like behavior.

## 1 Introduction

### 1.1 Overview of migraine

Migraine is a complex neurological disease that affects 15% of the adult population, and 7–11% of children and adolescents, making it one of the most common pain disorders (1–3). In both episodic and chronic forms, migraine dramatically affects an individual’s quality of life with moderate to severe attacks of unilateral pulsating pain, nausea, photophobia, and phonophobia (1, 4). Migraine attacks are often associated with four phases; a premonitory period, aura, headache, and a postdrome period (5). All four phases may not be present during a single attack. In the first phase, premonitory symptoms include food cravings, mood fluctuations, uncontrollable yawning, fluid retention, and increased urination (6–8). Premonitory symptoms can take place up to 24 h prior to the migraine attack and sometimes are followed by aura. During aura, individuals may experience visual disturbances such as flashing and bright lights (9–11). Headache is the phase in which pulsating pain (usually unilateral) is reported (12). Individuals may experience a postdrome phase with symptoms of neck stiffness, fatigue, and difficulty with concentration (13, 14). In addition to disabling symptoms, migraine presents an economic burden on society. In the United States, indirect costs associated with migraine are estimated at roughly $19 billion (15).

While migraine occurs in both children and adults, migraine has a typical onset in adolescence and is predominant in women (1). Migraine is three times more prevalent in women than men, but the definitive cause of this difference is not fully understood (16). The sexually dimorphic prevalence in migraine is commonly attributed to fluctuating levels of sex hormones associated with menstruation, pregnancy, and menopause (17–19). Several women experience more frequent migraine attacks around their menstrual periods, during the first trimester of pregnancy, and in perimenopause (20, 21). Questions remain if sex hormones are the sole reason for the disproportionality of migraine cases in women compared to men. The main objective of this review was to investigate preclinical literature on the effect of CGRP agonism and antagonism on migraine-like behavior; highlighting the differences that are observed between male and female rodents.

### 1.2 Migraine management

Treating migraine remains a challenge. Both non-prescription analgesics (e.g., acetaminophen and nonsteroidal anti-inflammatory drugs) and migraine-specific medications (e.g., ergots and triptans) have been shown to be effective for acute treatment of symptoms associated with migraine (22–24). Over-the-counter analgesics and nonsteroidal anti-inflammatory drugs (NSAIDs) are reported as being a convenient first-line therapy for mild-to-moderate migraine attacks (25). NSAIDs (e.g., ibuprofen) block cyclooxygenase enzymes (COX 1 and COX 2) that break down arachidonic acid into prostaglandins (26). Through the inhibition of COX-2, NSAIDs block inflammatory mediators that are thought to be upregulated during migraine and pain (26). Chronic blockade of COX-2 has been associated with an increased risk of heart attacks, and chronic blockade of COX-1 can lead to stomach ulcers, making NSAIDs unfavorable long-term (26). Ergots (e.g., dihydroergotamine and ergotamine) are thought to alleviate migraine symptoms, but the mechanism is not well understood. However, the use of ergots is limited due to their low degree of receptor activity and specificity (26). Ergots target a broad range of receptors (e.g., 5HT_1A_, 5HT_2A_, 5HT_1F_, 5HT_2C_, 5HT_3_, and D1 and D2 dopamine receptors) contributing to many adverse side effects, including nausea, distal muscle cramps, and angina (26, 27). Ergots are not recommended for patients with any coronary disease or pregnancy (26).

Triptans are agonists of serotonin receptors (5-HT_1B_ and 5-HT_1D_) often prescribed to treat symptoms associated with migraine (22, 28). Although triptans have a more desirable side effect profile than ergots, their use is associated with adverse side effects such as chest and/or neck pain, dizziness, and fatigue (23, 29). Also, over time, both triptans and ergots have been shown to sometimes worsen the frequency of headaches and produce medication overuse headache (MOH) (30). Ditans are a newer class of medication for acute migraine relief (31). Lasmiditan (i.e., Reyvow) is currently the only FDA-approved ditan. Lasmiditan is a potent and selective agonist of the 5-HT_1F_ receptor that has been shown to alleviate migraine symptoms by inhibiting activation of neurons in the trigeminal nucleus caudalis and by inhibiting the release of CGRP in peripheral and central trigeminal nerve terminals (32, 33). Unlike triptans, which are thought to mainly work by constricting blood vessels, ditans’ lack of vasoconstrictive properties makes them suitable for patients with cardiovascular risks (24, 34). Taken together, there is a pressing need to discover new therapeutics for migraine treatment. In the last two decades, drugs targeting calcitonin gene-related peptide (CGRP) signaling have been developed and approved for acute and preventative treatment for migraine.

### 1.3 Calcitonin gene-related peptide in migraine

Although the exact cause of migraine is not entirely understood, the trigeminovascular system is thought to play a critical role in migraine (35). CGRP is the most abundant neuropeptide in the trigeminal nerve and is believed to be released in the trigeminal system during migraine attacks (1, 36). CGRP consists of two isoforms, CGRPα and CGRPβ, which differ by three amino acids in humans (37). CGRPα is found primarily in the central and peripheral nervous system, showing high levels of expression in the trigeminal ganglion (38, 39). In comparison, CGRPβ is found mainly in the enteric nervous system (37). The CGRP receptor consists of three proteins, calcitonin receptor-like receptor (CLR), receptor activity modifying protein (RAMP), and receptor component protein (RCP), that form the CGRP receptor complex (37). CLR is a seven-transmembrane GPCR that is coupled to Gαs subunit (37). CLR is attached to the single transmembrane domain protein RAMP1 (37). This binding helps present the receptor at the surface of the plasma membrane and provides ligand specificity (37). RCP is an intracellular membrane protein that helps the RAMP1 and CLR heterodimer couple to the Gαs subunit (37). RCP primarily helps optimal facilitation of the Gαs signal transduction pathway rather than the binding of the CGRP peptide itself (37). The extent of receptor expression is not fully understood but is accepted to be unevenly expressed throughout the nervous system (1). Upregulation of RAMP1 expression increases CGRP sensitivity in the trigeminal ganglia, which could have implications for migraine (1).

CGRP is a well-known marker of “peptidergic” nociceptive neurons in mouse and rat sensory dorsal root ganglion (DRG) as well as trigeminal ganglion (TG). CGRP has been found to impact nociception, inflammation, and vasodilation (40). High levels of CGRP are found in blood plasma and saliva during migraine attacks, and injection of CGRP can lead to migraine-like attacks in individuals with chronic migraines (37). Modulation of CGRP signaling is associated with anti-migraine medicines, including small molecule receptor antagonists and monoclonal antibodies. Currently, there are four monoclonal antibodies approved by the FDA on the market: erenumab, eptinezumab, fremanezumab, and galcanezumab (Table 1). Erenumab was the first one of these drugs to be approved in 2018; it works as a monoclonal antibody against the CGRP receptor (41). Eptinezumab, fremanezumab, and galcanezumab are monoclonal antibodies for the CGRP neuropeptide itself rather than the receptor (37). In addition, there are three receptor antagonists (rimegepant, ubrogepant, and zavegepant) now available (Table 1).

**Table 1:** FDA approved CGRP-targeting monoclonal antibodies and antagonists for migraine.

Brand Name	Generic name	Other names	Developers/licensing company	Administration	Maximum dosing	Approval date	Class
Aimovig	Erenumab	AMG-334	Amgen Inc. and Novartis Pharmaceuticals	SC	140 mg/30 days	2018	CGRP receptor mAb/inhibitor
Vyepti	Eptinezumab	ALD403, ALD405	Lundbeck Seattle Biopharmaceuticals	IV	300 mg/90 days	2020	CGRP mAb/inhibitor
Ajovy	Fremanezumab	TEV-48125	Rinat Neuroscience, Pfizer, and Teva	SC	225 mg/30 days	2018	CGRP mAb inhibitor
Emgality	Galcanezumab	LY2951742	Eli Lily and Company	SC	240 mg* 120 mg/30 days	2019	CGRP mAb inhibitor
Nurtec	Rimegepant	BHV-3000, BMS-927711	Biohaven Pharmaceutical Holding Company	ODT	75 mg/24 h	2020	CGRP receptor antagonist
Ubrelvy	Ubrogepant	MK-1602	Merck & Co and Allergan	ODT	200 mg/24 h	2019	CGRP receptor antagonist
Zavzpret	Zavegepant	BHV-3500	Pfizer	Intranasal	10 mg/24 h	2023	CGRP receptor antagonist

SC, subcutaneous; IV, intravenous; ODT, orally disintegrating tablet. * The loading dose of emgality is 240 mg SC (2 consecutive 120 mg SC injections) and the maintenance dose is 120 mg per month. Dosing values are for human clinical use.

Although these therapies have been on the market for only half a decade, several studies have already been published demonstrating their safety and efficacy (42–45). These drugs are indicated for individuals with either chronic or episodic migraines. Chronic migraine is classified as having more than 15 headaches per month where at least 8 days meet the criteria for migraine, and episodic is characterized by fewer than 15 headaches per month. While most clinical trials report male and female numbers in research participants, very few studies stratify the data by sex or gender (46). This is particularly surprising given the significant sex differences reported in migraine prevalence. A recent systematic review by Alonso-Moreno et al. (47) highlights gender bias in published clinical trials on CGRP monoclonal antibodies authorized for prophylactic treatment of migraine. Researchers concluded that only 2 of the 25 studies included a sex-based analysis of the primary endpoint (48, 49). This is largely due to the low proportion of men who participate in clinical trials, leading to low power to detect sex differences. A recent evaluation by Porreca et al. (50) reported that for acute migraine treatment, small molecule CGRP-R antagonists only appear to be effective in women, while CGRP-R targeting antibodies are effective for migraine prevention in both male and female episodic patients. Overall, sex differences in CGRP’s role in migraine have been underexplored (51). The primary objective of this narrative review is to highlight preclinical studies examining the effects of CGRP in rodents, with the aim of exploring potential sex differences reported in the literature.

### 1.4 Rodent models of migraine

Rodent models of migraine have played a critical role in advancing the understanding of the pathophysiology of migraine and testing potential therapeutic interventions. Their contribution lies in the ability to mimic various aspects of the human migraine experience, such as mechanical allodynia, facial expressions of pain (e.g., grimace), photophobia, and decreased locomotor activity. Common animal models of migraine include dural applications of inflammatory agents (e.g., inflammatory soup/cocktail) (52–54), administration of nitric oxide (NO) donors and vasodilators (e.g., NTG) (55–57), stress induction (58–60), and repeated administration of acute migraine treatments (e.g., MOH) (61).

Inflammatory agents and NO donors are believed to induce migraine-like behavior by activating and sensitizing trigeminovascular afferents, promoting vasodilation of blood vessels, triggering neurogenic inflammation, and releasing other neuropeptides linked to migraine (62–65). Commonly used inflammatory agents (e.g., histamine, serotonin, bradykinin, prostaglandin E_2_), capsaicin, and complete Freund’s adjuvant (CFA) have been used to model migraine in rodents when applied to the dura singly or in combination as an inflammatory soup/cocktail (54). Nitric oxide donors such as nitroglycerin (NTG), have been reported to induce CGRP secretion in trigeminal ganglia neurons (63). Additionally, CGRP levels are elevated in the blood plasma of patients during an NTG-induced headache attack (66). Repetitive administration of inflammatory agents or NO donors has been shown to be an appropriate rodent model of chronic migraine and recurrent headache (54).

Stress is among the most common contributors to migraine attacks (67). Therefore, various stress models have been used to induce migraine-like behaviors in rodents. Researchers have utilized both acute and chronic stress paradigms, such as restraint stress, social defeat stress, early life stress, bright light, unpredictable sound stress, or a combination of different stressors (58–60, 68–71). Although there is evidence of a correlation between stress and migraine, the mechanisms linking the two are not fully known. Stress is thought to induce migraine-like behaviors through various mechanisms, including increasing NO synthase expression (72), altering the levels of various neurotransmitters and hormones that activate the trigeminovascular system (68, 73), decreasing the threshold for cortical-spreading depression (74, 75), and hyperalgesic priming (59).

Frequent or excessive use of medications such as opiates, barbiturates, NSAIDs, as well as medications intended to treat acute headache symptoms, can often lead to MOH, where patients experience an increase in headache frequency and intensity (76, 77). In preclinical research, MOH is modeled in rodents through repeated administration of medications such as triptans (61, 78, 79), ditans (80), and NSAIDs (81, 82), leading to behavioral and physiological changes that resemble the human condition. Several mechanisms have been proposed to underlie MOH, including cortical spreading depression (83), central sensitization (62, 84, 85), dysregulation of the descending pain-modulatory pathway (86, 87), and neuroinflammatory changes in the trigeminovascular system (88). Some preclinical evidence suggests that CGRP may be involved in the pathogenesis of MOH, as repeated triptan administration has been shown to result in an increase in CGRP labeling in trigeminal dural afferents (78). All together, many models of migraine are used to better understand the pathology of migraine, many of which will be highlighted in this review.

## 2 Methods

### 2.1 Search strategy

Published primary literature was identified using the PubMed database.^1^ The following terms were used to identify sources: “CGRP AND Migraine AND animal models NOT (Review[Publication Type])” on searches conducted up until February 2025. 131 papers were found and evaluated for inclusion in this narrative review. Since our focus was on behavioral outcomes, studies were excluded from the review when the only outcomes were molecular in nature. Overall, we focused our review on 35 studies (58, 59, 61, 71, 89–119). Studies were evaluated based on whether experiments focused on CGRP-induced migraine-like behavior or CGRP antagonist treatment of migraine models in animals. In some circumstances, single studies evaluated both CGRP and CGRP antagonists.

### 2.2 Risk of bias analysis

**Figure 1:**
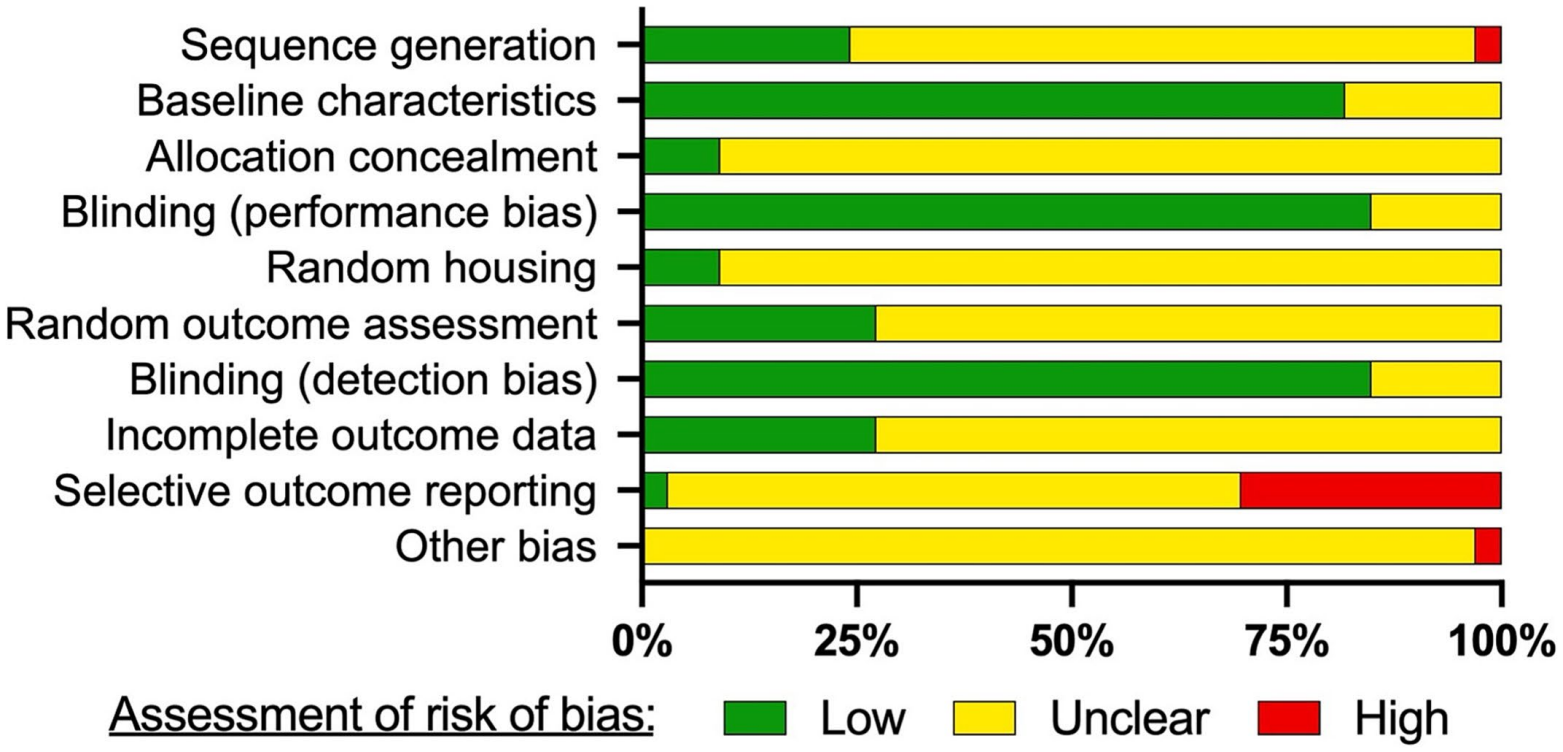
Risk of bias assessment. The SYRCLE’s risk of bias tool for animal studies was used to assess the risk of bias for each study included in the review. The risk of bias is presented as a percentage of the 35 included studies. Articles were evaluated according to the following domains: (1) sequence generation: was the allocation sequence adequately generated and applied? (2) baseline characteristics: were the groups similar at baseline or were they adjusted for confounders in the analysis? (3) allocation concealment: was the allocation adequately concealed? (4) blinding (performance bias): were the caregivers and/or investigators blinded from knowledge which intervention each animal received during the experiment? (5) random housing: were the animals randomly housed during the experiment? (6) random outcome assessment: were animals selected at random for outcome assessment? (7) blinding (detection bias): was the outcome assessor blinded? (8) incomplete outcome data: were incomplete outcome data adequately addressed? (9) selective outcome reporting: are reports of the study free of selective outcome reporting? (10) other sources of bias: was the study apparently free of other problems that could result in high risk of bias? (e.g., a wide range of n values across groups). Each type of bias classified as low, medium, or high risk of bias.

To evaluate the impact of experimental rigor in our review, we conducted a risk of bias analysis on the 35 studies included in this review. We assessed the risk of bias in accordance with the SYRCLE’s risk of bias tool for animal studies (120). Similar to The Cochrane’s risk of bias (RoB) tool (121), SYRCLE’s tool is typically used to assess the risk of bias in randomized controlled trials (RCTs). Articles were evaluated according to the following domains: (1) *sequence generation:* description of methods used to generate the allocation sequence of groups, (2) *baseline characteristics:* describing and/or testing if experimental and control groups were similar at baseline, before intervention, (3) *allocation concealment*: describe the method used to conceal the allocation sequence, (4) r*andom housing:* describe whether or not animals were housed randomly within the animal room, (5) *random outcome housing:* describe whether or not animals were selected at random and which methods were used, (6) *blinding (performance and detection bias)*: describe if caregivers and/or researchers were blinded to intervention and was the intended blinding effective, (7) *incomplete outcome data:* describe if incomplete outcome data was adequately addressed (e.g., exclusions), (8) *selective outcome reporting:* describe whether or not reports of the study are free of selective outcome reporting, and (9) *other sources of bias:* any important concerns about bias not covered by other domains in the tool (e.g., a wide range of n values across groups). Each domain was determined as “low risk,” “unclear risk,” and “high risk” (Figure 1). For analyzed data, risk of bias analysis was completed using SYRCLE’s bias tool for animal studies to provide summary assessments of the risk of bias (Figure 1). An unclear risk of bias was observed in many of the domains. Sequence generation, allocation concealment, random housing, random outcome assessment, incomplete outcome data, selective outcome reporting, and other bias had unclear risk of bias in over 50% of the included studies. Low risk of bias was found in ~75% of the included studies for blinding (performance and detection bias) and for baseline characteristics. In ~30% of the included studies, high levels of selective outcome reporting were present. This is due to some data not being shown in the included articles.

## 3 Effect of CGRP on migraine behavior

**Figure 2:**
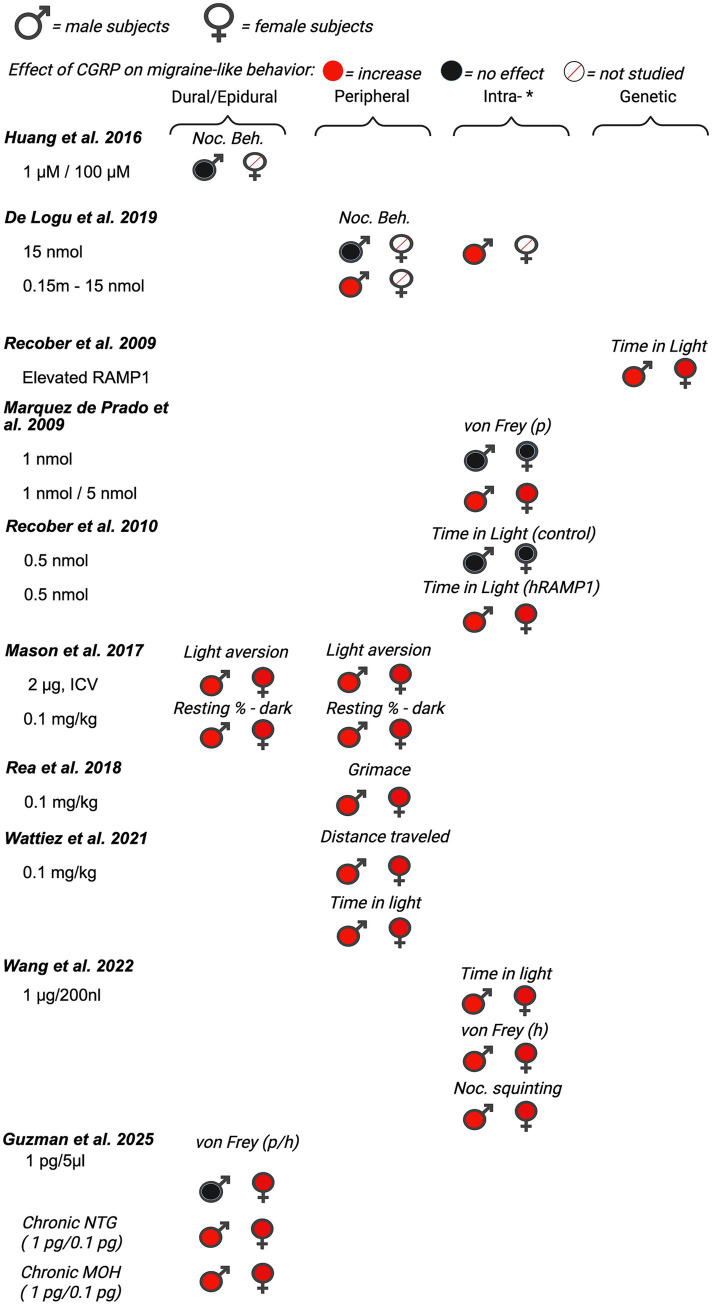
Summary of sex differences in studies assessing the role of CGRP on migraine-like behavior in mice. Studies are categorized by route of administration of CGRP (dural/epidural, peripheral, *intra-[intrathecal, intracisternal, intracerebroventricular, intraganglionar], and transgenic mouse model [nestin/hRAMP1]). Black, filled symbols indicate CGRP agonism had no effect on migraine-like behavior. Red, filled symbols indicate CGRP agonism increased migraine-like behavior. A red, diagonal line through the symbol indicates that CGRP agonism was not assessed in that particular sex. (p) = periorbital, (h) = hindpaw.

**Figure 3:**
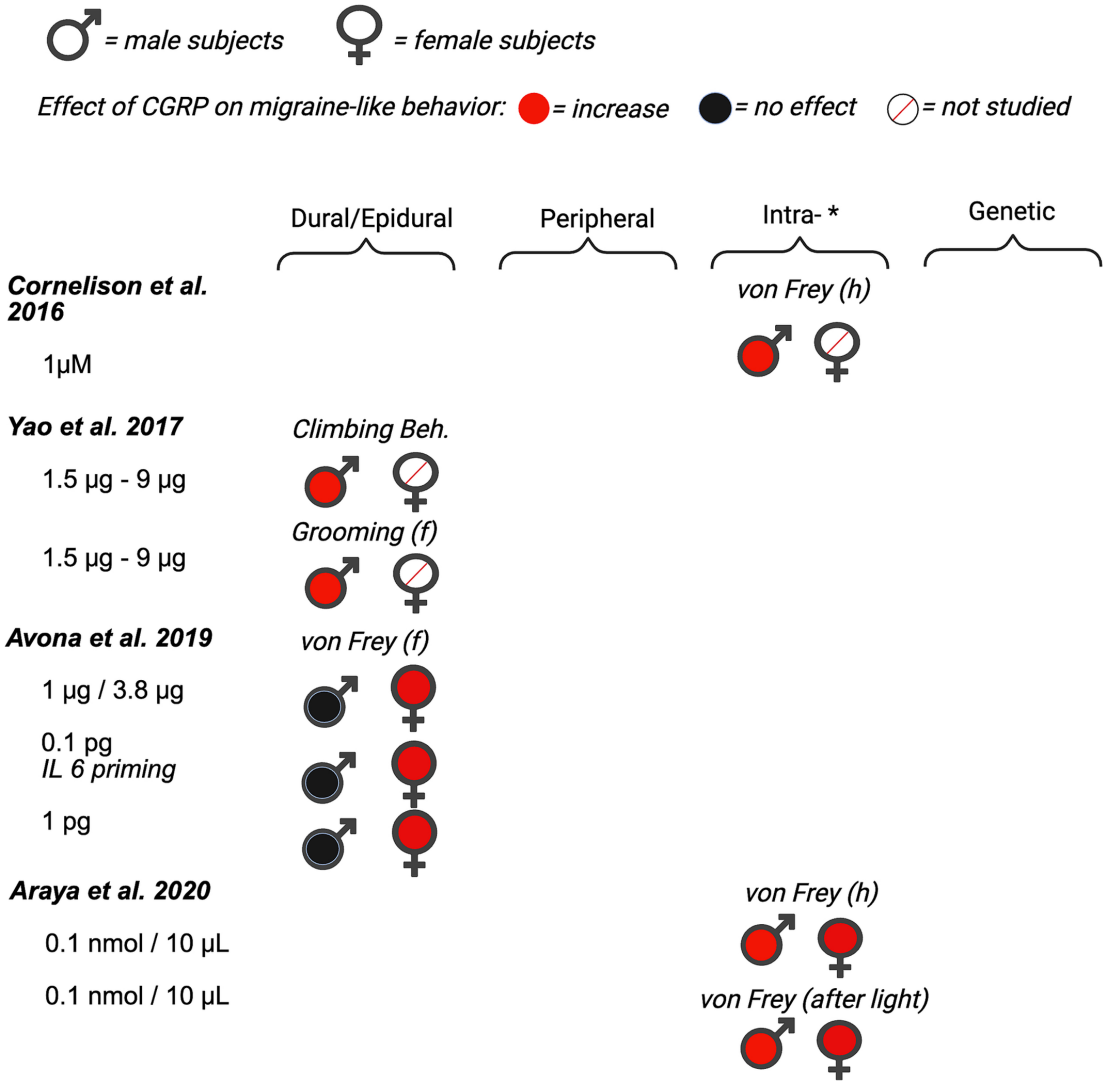
Summary of sex differences in studies assessing the role of CGRP on migraine-like behavior in rats. Studies are categorized by route of administration of CGRP (dural/epidural, peripheral, *intra-[intrathecal, intracisternal, intracerebroventricular, intraganglionar], and transgenic mouse model [nestin/hRAMP1]). Black, filled symbols indicate CGRP agonism had no effect on migraine-like behavior. Red, filled symbols indicate CGRP agonism increased migraine-like behavior. A red, diagonal line through the symbol indicates that CGRP agonism was not assessed in that particular sex. (f) = face/facial, (h) = hindpaw.

First, we evaluated 14 of the 35 manuscripts that measured the direct effect of CGRP on migraine-like behaviors in rodents. Pain is subjective by nature, making it challenging to assess it in preclinical rodent studies. Many models of migraine and different assays of migraine-like behavior in rodents have been reviewed as summarized above, with each presenting its own set of limitations (122, 123). Migraine-associated behaviors included in the review were von Frey mechanical sensitivity, light–dark assays, spontaneous nocifensive behavior, ultrasonic vocalizations, facial grimaces, hypoactivity, and immobility time. Nocifensive behavior is characterized by grooming or scratching of the body. Of the 14 manuscripts, four were conducted in only male rodents and 10 used both male and female rodents within a single study although data were not always stratified by sex in those studies. The reviewed articles are organized below based on the date of initial publication and summarized in Figure 2 (data from mice), Figure 3 (data from rats), and Supplementary Table 1.

### 3.1 Effect of CGRP on migraine behavior in male rodents

Although migraine is primarily prevalent in women, several studies investigating the effect of CGRP on migraine-associated pain have been conducted in male rodents. Huang et al. (89) investigated the effect of dural application of CGRP on nocifensive behavior in male Swiss Webster mice. In this study, nocifensive behavior was scored by measuring the time spent on forepaw wiping and hindpaw scratching within the mouse V_1_ dermatome (i.e., scalp and periorbital area). Nocifensive behavior was examined after the application of both 1 μM and 100 μM of CGRP dissolved in aCSF. Neither the 1 μM nor the 100 μM CGRP applied on the dura significantly increased nocifensive behavior in naïve (pain-free) mice. Huang et al.(89) also observed the effects of CGRP antagonist CGRP_8-37_ on nociceptive behavior in the context of an inflammatory model of migraine. Evaluation of CGRP_8-37_ effects in this manuscript is shown below in the review of CGRP inhibition studies (See Section 4.1).

Cornelison et al. (90) evaluated the effect of increased CGRP levels in the spinal cord on primary nociceptive sensitivity. The researchers explored the peripheral sensitization of trigeminal ganglion nociceptors by co-injecting CGRP with a receptor antagonist (CGRP_8-37_), or co-injecting CGRP with an inhibitor of protein kinase A (KT 5720) (90). This experiment used the same dose of CGRP (1 µM) as the investigation by Huang et al. (89), but in this study CGRP was injected intracisternally in the upper cervical spinal cord in adult male Sprague–Dawley rats. Nocifensive head withdrawal responses to a von Frey filament (100 g) were measured 2 h and 1–3 days post-injection. An increase in nocifensive head withdrawal was observed 2 h, 1 day, and 2 days post-injection of CGRP when compared to saline (naïve) controls. This effect was blocked when CGRP was co-injected with CGRP_8-37,_ suggesting specificity for the *Calcrl* receptor. The results of this study support the notion that CGRP-mediated central sensitization leads to an increase in trigeminal nociceptor sensitivity, suggesting that central-to-peripheral signaling helps explain how peripheral nociceptors become sensitized.

Yao et al. (91) established a novel rat model of migraine by epidural injection of CGRP to resemble the clinical symptoms of migraine patients. In this study, male Sprague–Dawley rats received epidural injections of CGRP (1.5, 3, 6, or 9 mg) or normal saline (20 mL). Migraine pain-like behaviors were analyzed every 30 min during the 240 min experiment. The pain behaviors that were investigated included cage climbing (climbing hutch), facial-grooming, body-grooming, facial grooming with the ipsilateral hindpaw, resting behavior, freezing, and immobility (91). We focused on climbing behavior, facial grooming, and immobility, as no effects were reported for other measures. There was a significant dose–response relationship for climbing behavior, facial grooming, and immobility, indicating that higher doses of CGRP induced the strongest responses, with the lowest dose failing to elicit significant migraine-like behavior for climbing and immobility. The authors’ concluded that this model could be used for studying new drug candidates for migraine treatment. This study also highlights the necessity of testing a broad range of CGRP doses when comparing different behavioral endpoints.

De Logu et al. (92) investigated different endogenous mediators to determine which mediators elicit delayed and prolonged periorbital mechanical allodynia. They tested CGRP, adrenomedullin, histamine, amylin, prostaglandin E_2_ (PGE_2_), prostacyclin (PGI_2_), prostaglandin F_2α_, and pituitary adenylyl cyclase activating peptide (PACAP). Male C57BL/6 J mice were injected subcutaneously in the periorbital area with different doses of CGRP (p.orb., 10 ul/site). Following injection, spontaneous nociception and periorbital mechanical allodynia were recorded and measured. Spontaneous nociceptive behavior was defined as the duration spent rubbing the face (i.e., the injection site) (92). 15 nmol of CGRP had no effect on face rubbing (i.e., nocifensive behavior). This is similar to the study conducted by Huang et al. (89), where CGRP had no effect on nocifensive behavior in male mice. However, significant pronociceptive effects were seen in the facial von Frey assay after periorbital injections of 0.15 nmol, 1.5 nmol, and 15 nmol of CGRP 1 h, 2 h and 4 h post-injection. Suggesting that while CGRP results in both nocifensive and mechanical allodynia in rats, male mice display mechanical allodynia in response to CGRP, but not other nocifensive behavior. In addition to CGRP, other endogenous mediators (PACAP, PGE_2_, PGI_2_) also evoked dose-dependent periorbital mechanical allodynia. Taken together, the results from this study support the ability of migraine-provoking substances to initiate mechanical allodynia by acting on peripheral terminals of trigeminal afferents.

### 3.2 The effect of CGRP on migraine behavior in both male and female rodents

Of the 14 papers that studied the effects of CGRP on migraine-like behavior, 10 investigated the effects of CGRP on migraine-like behavior in both male and female rodents. Of these Recober et al. (93) sought to determine whether heightened CGRP sensitivity leads to migraine-associated symptoms in mice. They studied light aversive behavior in migraine by using a transgenic mouse line (*nestin/hRAMP1*), which upregulates the receptor protein RAMP1, a subunit of the CGRP receptor required for CGRP binding (93). The primary behavioral assay employed in this experiment was the light-aversion test, which was quantified by measuring the time the mouse spent in the light versus the dark compartment. A decrease in time spent in light is regarded as photophobia (i.e., light-aversion), a symptom commonly reported in migraineurs (35, 124). Both male and female *nestin/hRAMP*1 mice showed significant migraine-like behavior when compared to control littermates. Although not significant, female *nestin/hRAMP*1 mice displayed more photophobia (less time in light) than male *nestin/hRAMP*1 mice. Since no significant sex differences were observed in this initial experiment, the authors reported that both sexes were pooled for the remaining tests in the manuscript. In the combined group, they tested the effect of intracerebroventricular CGRP (0.5 nmol), CGRP with olcegepant, formally known as BIB409BS (0.5 nmol each), or vehicle on light-aversive behavior in mice. *Nestin/hRAMP1* male and female pooled mice treated with CGRP displayed significantly more light-aversive behavior than control littermates and *nestin/hRAMP1* mice treated with vehicle. This effect was observed in the 1^st^ and 2^nd^ 300-s intervals of the test. The effect of CGRP on light-aversive behavior was prevented when CGRP was administered with CGRP receptor antagonist – olcegepant.

Marquez de Prado et al. (93) sought to study the role of CGRP in mechanical allodynia. Similar to the previous study, this study also used male and female *nestin/hRAMP1* mice, as well as control littermates (94). Data from male and female mice were pooled and not analyzed by sex. Male and female *nestin/hRAMP1* mice or control mice received CGRP intrathecally (1 and 5 nmol) or peripherally (1.3 nmol) 30–90 min and 5–60 min, respectively, prior to mechanical allodynia testing (via von Frey). Under baseline conditions, nestin/*hRAMP1* and control littermates displayed similar hindpaw mechanical nociception. Intrathecal CGRP (1 nmol) evoked hindpaw mechanical allodynia in male and female *nestin/hRAMP1 mice*, but not control mice, 30–60 min after treatment. This effect was blocked when CGRP was co-administered with the CGRP antagonist, CGRP _8–37_ (5 nmol). 5 nmol of CGRP was required to induce hindpaw mechanical allodynia in male and female control mice. In contrast to central administration of CGRP, intraplantar administration of CGRP failed to induce hindpaw mechanical allodynia in both *nestin/hRAMP1* and control mice. These data suggest that CGRP is acting centrally to induce mechanical hypersensitivity, but the potential for sex differences is unclear due to pooling across males and females.

Recober et al. (95) further characterized the photophobic phenotype of *nestin/hRAMP1* male and female mice in another report. Data from both male and female mice were pooled and authors did not report observed sex differences in behavior. They sought to measure and compare photophobic behavior from their previous study (93), in a smaller testing apparatus with a considerably lower light intensity. Additionally, in this follow-up study, Recober et al. were able to measure additional parameters such as transitions, latency to enter/exit the dark, rearing, resting, ambulatory distance, ambulatory time, and ambulatory velocity. *Nestin/hRAMP1* mice treated with central CGRP (0.5 nmol, intracerebroventricularly (ICV)) spent less time in the light compared with control mice and vehicle treatment. Additionally, CGRP-induced motility changes in both *nestin/hRAMP1* and control mice in the dark compartment. CGRP-treated *nestin/hRAMP1* mice displayed fewer rearings than vehicle-treated mice in the dark zone, but no effect was observed in the light zone.

Mason et al. (125) reported that CGRP can act in both the brain and the periphery to induce migraine-like symptoms in pooled male and female rodents. They studied the effect of peripheral CGRP (0.1 mg/kg, intraperitoneal (IP)) on two strains of mice; CD1 and C57BL/6 J mice. The authors report that peripheral CGRP induces light aversion in both strains as indicated by a decrease in the amount of time spent in the light zone during sequential exposures in the light/dark chamber. Researchers also studied the effect of peripheral CGRP on motility by measuring resting time, vertical beam breaks in the light and dark zone, and transitions (between the light and dark zones). In both strains of mice, intraperitoneal CGRP significantly increased the resting time in the dark zone and decreased rearing behavior (reduced motility). Additionally, mice treated with CGRP transitioned significantly less between light and dark zones compared to vehicle-treated mice. This is different from what was observed in the Marquez de Prado et al. (94) study, where peripheral CGRP did not induce migraine-like behavior. The authors report strain differences in that CGRP-induced light aversion and reduced motility were greater and more consistent in CD1 mice compared to C57BL/6 J mice. Pretreatment with a monoclonal CGRP antibody prevented CGRP-induced light aversion in the CD1 mice. Next, they studied the effect of centrally administered CGRP. Intracerebroventricular injection of CGRP resulted in a significant light aversive behavior, and mice showed an increase in resting behavior in the dark, but not the light zone, when compared to vehicle-treated mice. Mason and colleagues report that they did not detect a significant difference in the time spent in light between male and female mice, although the data are not shown disaggregated by sex. However, the authors do report a trend toward CD1 female mice spending less time in the light after receiving 0.5 mg/kg of CGRP compared to males.

Rea et al. (97) sought to characterize spontaneous pain in mice that received peripheral injections of CGRP. Prior to this study, CGRP had been shown to influence light aversion and nociceptive reflexes, but the effect of peripheral CGRP on spontaneous pain had not been tested in mice (96, 126). Researchers studied the differences in grimace responses between male and female CD1 mice when given an intraperitoneal (IP) injection of 0.1 mg/kg CGRP (97). CGRP induced a significant pronociceptive effect that began 10 min after injection, lasting for 60 min. Although no significant differences were observed between males and females, it was apparent that a greater mean grimace score was observed in the female mice. Next, the authors looked at the effects of pretreatment of PBS (vehicle), meloxicam (2 mg/kg IP), sumatriptan (0.6 mg/kg IP), control antibody, and CGRP antibody ALD405 (30 mg/kg IP), on CGRP-induced spontaneous grimace. Regardless of pretreatment, all mice received CGRP (0.1 mg/kg IP) 30 min before testing. Meloxicam and the control antibody failed to block CGRP-induced spontaneous grimace in either sex. ALD405 pretreatment significantly attenuated the effect of CGRP in both males and females. A sex-specific response to treatment was observed with the antimigraine drug sumatriptan, which partially blocked the CGRP response in male but not in female mice. Next, the authors sought to see if CGRP-induced grimace behavior was light-dependent. Using a restrained mouse grimace scale assay, an increase in grimace behavior was observed in both the dark and light in two strains of mice. Researchers conclude that regardless of light, CGRP can still induce spontaneous pain and note the need for further studies evaluating the sex-specific responses.

Avona et al. (98) investigated sex differences of CGRP application and its implications on pain behavior in male and female Sprague Dawley rats and ICR mice. Researchers measured both hind paw and facial von Frey and grimace scores to evaluate migraine pain. Among males, there was little evidence of CGRP inducing pronociceptive effects measured with von Frey either with dural injection or peripheral injection. In contrast, for females, dural CGRP with or without IL-6 or BDNF priming produced robust pronociceptive effects. The 3.8 *μ*g dose of CGRP had the largest effect size, and 1.0 pg. CGRP had the lowest effect size on facial von Frey behavior. Paw injection of CGRP in females caused significant hypersensitivity at the paw only. There was a significant nociceptive effect of CGRP (100 *μ*M) on facial von Frey sensitivity but no significant effect on grimace scores compared to vehicle-treated male mice. Researchers note that this was the first evidence that CGRP-induced headache-like behavioral responses when applied to the dura at doses up to 3.8 *μ*g are female-specific both acutely and following central and peripheral priming. These data support a model where dural CGRP-based mechanisms contribute to the sexual disparity of migraine.

Araya et al. (99) investigated whether intraganglionic CGRP induced differential migraine-like responses, including periorbital mechanical allodynia, light sensitivity, and anxiety like behavior in rats. The first objective of this study was to test the effect of CGRP on periorbital allodynia in male and female Wistar rats. Either CGRP (0.1 nmol/10 μL) or saline was injected into the right trigeminal ganglion of rats that were pretreated with either vehicle or sumatriptan (1 mg/kg IP). Intraganglionar CGRP produced a pronociceptive response (decrease in mechanical thresholds), when compared to saline groups, in both female and male rats. However, female rats displayed a greater degree of periorbital allodynia that lasted for a longer duration than male rats. Sex differences were not observed in rats that were given intraganglionar CGRP following sumatriptan treatment. Next, the authors studied the effect of intraganglionic CGRP injection on photosensitivity. 24 h after CGRP treatment, animals were exposed to an aversive light and periorbital mechanical allodynia was measured until mechanical thresholds returned to baseline. Female rats displayed more prolonged periorbital mechanical allodynia following aversive light when compared with male rats. Significant periorbital mechanical allodynia was observed 1 h after light exposure in males and 1-4 h in females.

Araya and colleagues also studied the effect of intraganglionar CGRP on anxiety-like behavior in male and female rats. CGRP reduced the number of entries and time spent in the open arm of the elevated plus maze. Female rats treated with CGRP displayed significantly more time in the closed arms than saline controls, which is a anxiogenic response. Whereas, in male rats, a statistical difference was not observed in the closed arms between saline and CGRP-treated males. Researchers concluded that intraganglionar CGRP may play a major role in migraine-like responses such as periorbital mechanical allodynia, light sensitivity, and anxiety like-behavior. This study supports the understanding of sexual dimorphic CGRP signaling, as researchers note the difference in the prevalence of migraines between male and females and conclude that female rats are likely to be more susceptible to these effects.

Rodents are nocturnal yet the majority of animal studies are done during the day (inactive phase) due to convenience. Several studies report early morning or late night peaks of onset in migraine attacks in migraineurs (127–135). Wattiez et al. (100) used male and female outbred CD1 mice to compare the effect of peripheral CGRP (0.1 mg/kg IP) administration on migraine-like symptoms throughout the circadian cycle. Mice were injected with vehicle (PBS) or CGRP (0.1 mg/kg IP) each week over 3 consecutive weeks. In the light–dark preference, a decrease in time spent in light was regarded as a pronociceptive response; mice were tested twice on subsequent days (trial 1 vs. trial 2) (100). Generally, CGRP-induced reductions in time spent in the light in both male and female animals. However, the order of testing of trials (light phase before dark phase vs. dark phase before light phase) did have a subtle effect. In males, when trial 2 was in the light phase, CGRP no longer reduced the time spent in light (10 AM Test 2). In females, when trial 1 was in the light phase, CGRP did not reduce time in light (10 am Test 1). CGRP reduced time in the light during the dark phase in males and females irrespective of trial order. For the squint assay, a decrease in mean pixel area was quantified as a pronociceptive response to injection of CGRP vs. vehicle (PBS). There was a significant pronociceptive effect of CGRP in both male and female mice compared to vehicle (mean pixel area). The phase of daylight (light vs. dark) did not affect squinting behavior for males or females. Additionally, Wattiez et al. conducted tests observing locomotor activity (open field and wheel running). Male mice only exhibited a significantly decreased distance traveled across the circadian day after CGRP delivery. For wheel running, there was a trend for reduced wheel running for the first hour after CGRP in both male and female mice. Only females at 8 pm (dark period) exhibited a significant reduction in wheel running. Researchers note that while similar results were observed in both male and female mice, the study was not powered to fully detect sex differences. Overall, the Wattiez et al. results are consistent with other studies De Logu et al. (92), Mason et al. (125), and Rea et al. (97) evaluating peripheral CGRP application showing pro-nociceptive effects in both sexes.

Wang et al. (101) tested whether the cerebellum, particularly the medial cerebellar nuclei (MN), might be a site of CGRP action. CGRP was directly injected into the right MN of C57BL/6 J male and female mice via cannula, followed by behavioral tests that assessed migraine-like symptoms. The behavioral tests used included the light/dark assay, open field assay, von Frey test, automated squint assay, and gait dynamic assay. For drug administration, mice were given either 𝛼-CGRP (1 μg) or 1X PBS (220 nL) as the vehicle through injection cannulas. Results found injection of CGRP into the MN induced light-aversive behavior and reduced motility under dim light similarly between sexes. Mice, regardless of sex, injected with CGRP spent less time in light during light/dark assay. In addition, CGRP-injected mice had fewer rearings, however data did not reach statistical significance likely due to the variability and small sample size of each sex. Transitions between dark and light zones were significantly decreased by CGRP for both sexes. Stride length did not change following injection of PBS or CGRP into the MN, indicating CGRP administration decreases motility without gait alterations. In addition, injection of CGRP into the MN induced anxiety-like behavior during open field assay and induced plantar tactile hypersensitivity in the contralateral hind paw for both sexes. However, sex differences were observed in the severity of the migraine-like symptoms. In the open field assay, CGRP-treated female mice displayed a greater reduction in time spent in the center compared to CGRP-treated male mice. In the von Frey test, CGRP-treated female mice displayed a greater magnitude of mechanical hypersensitivity in the contralateral hindpaw compared to CGRP-treated male mice. Mechanical hypersensitivity in the hindpaw ipsilateral to the CGRP injection was similar between males and females. In the automated squint assay, CGRP-treated females displayed nociceptive squinting behavior (decrease in mean pixel area), but not males. Overall, they found that several migraine-like symptoms could be induced by CGRP in the cerebellum, which supports the hypothesis that the cerebellum contributes to migraine pathogenesis. Researchers concluded that CGRP injection into the cerebellum is sufficient to induce migraine-like behaviors in mice but note the effects on anxiety, tactile hypersensitivity, and squinting behavior are more prominent in females.

Guzman et al. (61) studied sex differences of CGRP on migraine pain-like behavior by using both naïve mice and mouse models of chronic migraine (CM) and MOH. First, they assessed migraine-like pain behavior in response to supradural CGRP (1 pg) in naïve mice. Periorbital and hindpaw allodynia was measured 20, 40, 60, 120, and 180 min following treatment of either CGRP or vehicle in male and female mice. Only female mice, displayed significant migraine-like pain behavior in response to CGRP, when compared to vehicle-treated controls. There was an increase in number of CGRP-positive neurons in the ophthalmic V1 region of the trigeminal ganglion in female mice, when compared to male mice. Next, the researchers sought to study sex differences as migraine frequency increases by using models of CM and MOH. Following baseline periorbital tactile responses, female and male mice received a subthreshold, supradural injection of CGRP (0.1 pg. and 1 pg., respectively). On the following day, CM migraine-like pain was induced in female and male mice by repeated administration of NTG (10 mg/kg, IP) every other day for 9 days (control animals received saline). In a separate group of animals, MOH was modeled in female and male mice by repeated administration of sumatriptan (10 mg/kg, IP) for 9 consecutive days. Migraine-like behavior was assessed every other day for 17 days. In comparison to saline groups, repeated administration of nitroglycerin (NTG) elicited periorbital and hindpaw allodynia in both female and male mice, with tactile thresholds returning to baseline levels by day 17 (i.e., 8 days after NTG termination). In comparison to the vehicle groups, repeated administration of sumatriptan elicited a significant increase in migraine-like behavior in both female and male mice, with tactile thresholds returning to baseline on day 20 (i.e., 11 days after sumatriptan termination). In either model, there were no sex differences observed in the development or maintenance of migraine-like pain behavior. For both CM and MOH models, the subthreshold dose of CGRP (0.01 pg. in females and 1 pg. in males) elicited an increase in migraine-like pain behavior, with similar CGRP sensitization observed in both male and female mice. This increased sensitivity to CGRP is accompanied by an increase in CGRP positive cell bodies in the TGV1 in male and female mice. Altogether, findings from this study suggest that while supradural CGRP induces migraine-like behavior preferentially in female mice, mice of both sexes develop sensitivity to CGRP with increase in migraine frequency. These murine data have a direct correlation to what is seen in the clinic. In the clinic, while preventative CGRP targeting therapies are effective in both men and women with EM or CM, acute therapies targeting CGRP for episodic migraine have demonstrated greater efficacy in women (50, 136, 137).

Taken together, when comparing the effects of CGRP, there are general pronociceptive, light-aversive, immobility, and anxiety-like effects observed in both male and female rodents with stronger effects in females when the two sexes are directly compared. Collectively, data from these studies largely supports reports from humans showing a potentially greater role in female patients and a less robust role in male patients. These similarities then support the premise that mechanistic studies in animal models using CGRP as an inducer of behavioral phenotypes will continue to yield valuable insights into the etiology of migraine.

## 4 Effect of CGRP inhibitors on migraine behavior

**Figure 4:**
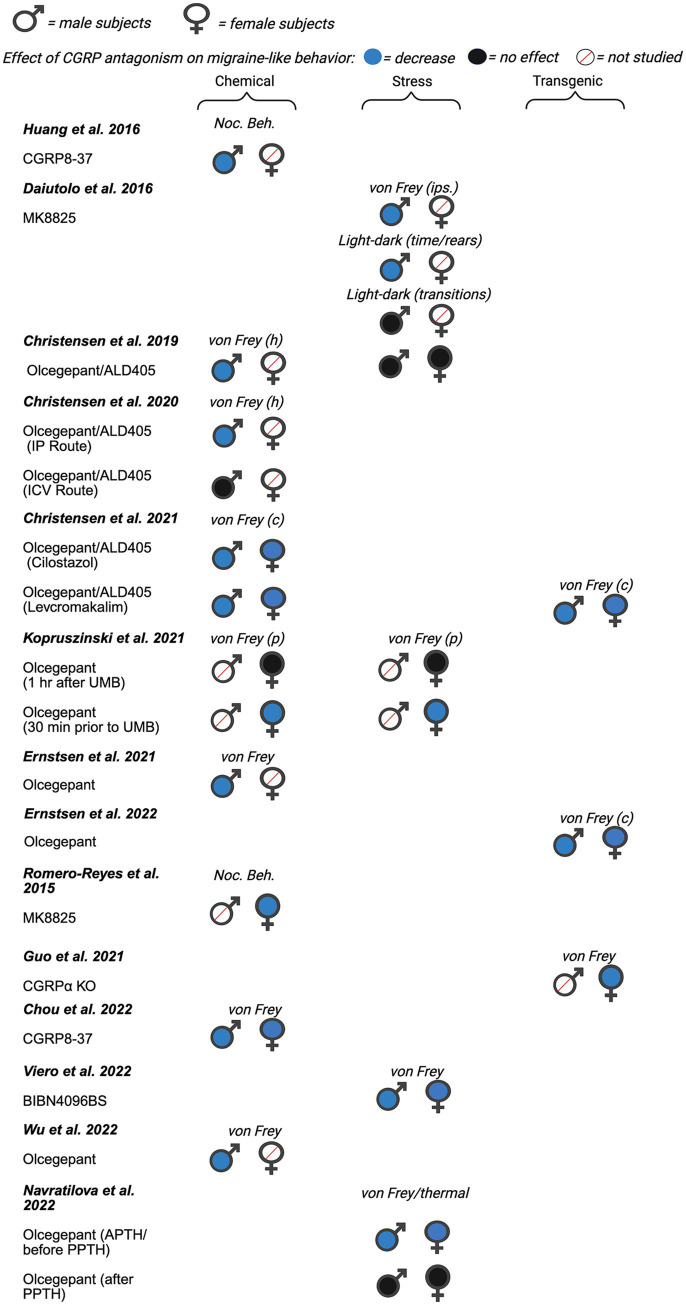
Summary of sex differences in studies assessing the role of CGRP antagonism on migraine-like behavior in mice. Studies are categorized by model of migraine/headache (chemical, stress, and transgenic). Black, filled symbols indicate CGRP antagonism had no effect on migraine-like behavior. Blue, filled symbols indicate CGRP antagonism decreased migraine-like behavior. A red, diagonal line through the symbol indicates that CGRP antagonism was not assessed in that particular sex. (ips.) = ipsilateral, (p) = periorbital, (c) = cephalic, (h) = hindpaw.

**Figure 5:**
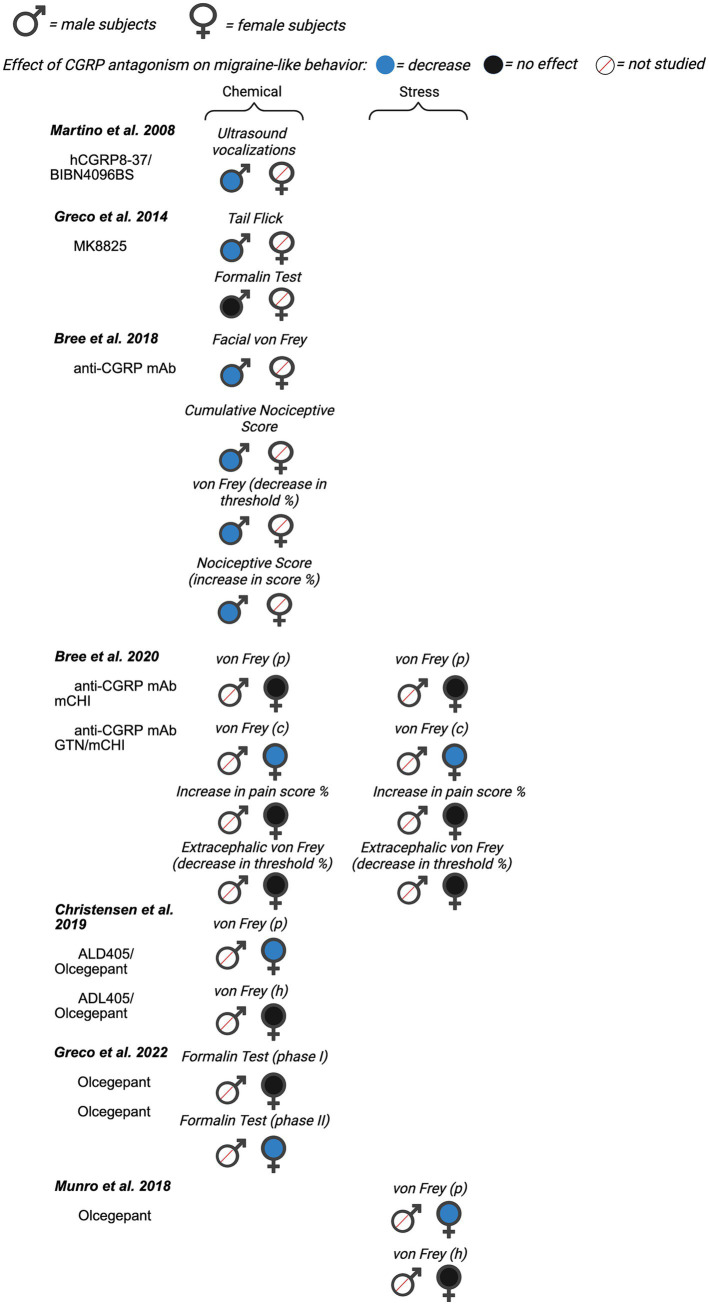
Summary of sex differences in studies assessing the role of CGRP antagonism on migraine-like behavior in rats. Studies are categorized by model of migraine/headache (chemical, stress, and transgenic). Black, filled symbols indicate CGRP antagonism had no effect on migraine-like behavior. Blue, filled symbols indicate CGRP antagonism decreased migraine-like behavior. A red, diagonal line through the symbol indicates that CGRP antagonism was not assessed in that particular sex. (p) = periorbital, (c) = cephalic, (h) = hindpaw.

Next, we evaluated the potential for sex differences in studies that inhibited CGRP signaling on migraine-like behavior. Of the 22 total papers focusing on CGRP antagonism, nine investigated male rodents only, six included females only, and seven studies included male and females within a single publication. A summary of all data from included manuscripts can be found in Figure 4 (data from mice), Figure 5 (data from rats), and Supplementary Table 2.

### 4.1 Effect of CGRP inhibitors on migraine behavior in male rodents

Martino et al. (102) sought to determine the validity of ultrasound vocalizations (USV) as a novel assay to assess the clinical efficacy of migraine therapies in rats. In this study, male adult Sprague Dawley rats received ICV injections of Lipopolysaccharide (LPS) to induce central inflammation (102). Numerous pharmacological agents known for their analgesic properties were used to test the face validity of the LPS-induced USV assay. These pharmacological agents included CGRP antagonists, hCGRP_8-37_ (2.5–50 nmol) and BIBN4096BS (olcegepant) (2–200 pmol). All doses of BIBN4096BS had a significant antinociceptive effect on USV, with the largest effect seen with 200 pmol administration and the smallest effect with 2 pmol. Both 50 nmol and 25 nmol of CGRP_8-37_ had significant antinociceptive effects, but no effect was seen with 2.5 nmol (102). Researchers concluded their model of tactile-induced USV following cerebral inflammation shows a pharmacology profile that is predictive of efficacy in migraines. Researchers note that further studies are necessary to understand the mechanisms in their model to correlate with known mechanisms in migraine.

Greco et al. (103) investigated the effect of CGRP antagonist MK-8825 (an analog of MK-3207) in a NTG-induced migraine model. They hypothesized that MK-8825 would produce analgesic effects in two pain assays, tail flick test (TFT) and the formalin test, during NTG-induced hyperalgesia of male Sprague–Dawley rats. Antinociceptive effects were classified as a decrease in total flinches and paw shaking for the formalin test and an increase in withdrawal latency for the tail-flick test. MK-8825 (10 mg/kg, IP) administered simultaneously, 30 min, and 60 min after NTG, significantly reduced total flinches and paw shakes in phase II (10–60 min after formalin) of the formalin test. MK-885 failed to show an analgesic effect in phase I. In the tail-flick test, MK-8825, which was administered simultaneously, 30 min and 60 min prior to NTG, displayed a significant increase in withdrawal latency. However, MK-8825, which was administered 3.5 h after NTG administration, failed to produce an antinociceptive effect (103). Overall, the researchers’ results suggest that MK-8825 may represent a potential therapeutic tool for treating migraines in males.

As mentioned previously, the study by Huang et al. (89) which evaluated the pronociceptive effects of CGRP also observed the effects of CGRP antagonist CGRP_8-37_ on nociceptive behavior. This research was completed in the context of a mouse model of headache using dural application of capsaicin and inflammatory mediators (IScap) (89). Researchers administered 1 μM of the CGRP receptor antagonist CGRP_8-37_ onto the dura (89). CGRP_8-37_ reduced IScap-induced nocifensive behavior in adult male Swiss Webster mice.

CGRP and nitric oxide synthase (NOS) are known to play a role in the pathology of post-traumatic headache (PTH). Daiutolo et al. (105) assessed the relationship between inducible nitric oxide synthase (iNOS) and CGRP in a model of controlled cortical impact (CCI) injury in male C57BL/6 J mice. They investigated the effect of Sumatriptan (a 5-HT1B/1D receptor agonist used to treat acute migraine headaches) and CGRP antagonist - MK 8825 (100 mg/kg IP) on migraine-like behavior. Migraine behavior was evaluated by testing trigeminal allodynia and photosensitivity via periorbital von Frey and light–dark box tests, respectively. Both sumatriptan and MK8825 significantly reduced CCI-induced trigeminal allodynia on the ipsilateral side, compared to vehicle-treated controls. Sumatriptan also reduced trigeminal allodynia on the contralateral side, but MK8825 did not. When assessing photosensitivity/photophobia, researchers tested different intensities of light (light:dark preference) and evaluated rearing behavior. Exploratory behaviors during bright light showed reduced rearing and light-avoidance behavior, indicative of photosensitivity, and rearing were improved by MK8825 treatment, but not sumatriptan. There was also a significant antinociceptive effect for the percent of time spent in bright light versus moderate light intensities when administered with MK8825. There was no significant effect in the number of rears or transitions in different light intensities with MK8825 administration. Overall, CGRP antagonism showed better efficacy for attenuating headache behaviors including trigeminal allodynia and photosensitivity, than sumatriptan.

Bree et al. (106) investigated treatment for migraine caused by mild post-traumatic brain injury (mCHI) in adult male Sprague–Dawley rats. A weight drop device was used to induce mCHI in rats. To measure tactile pain hypersensitivity, responses to von Frey filaments were tested at baseline, 48 h, 72 h, 7, and 14 days post mCHI. Researchers studied the effects of sumatriptan (1.0 mg/kg, IP) and chronic treatment of anti-CGRP mAb (30 mg/kg, IP) on tactile hypersensitivity. Anti-CGRP mAb was given immediately after mCHI and every 6 days subsequently. There was a significant reduction in tactile hypersensitivity and a trending antinociceptive effect on the cumulative nociceptive score after anti-CGRP mAb treatment in the context of mild closed head injury (mCHI only). Anti-CGRP mAb significantly attenuated mCHI-induced tactile hypersensitivity and the cumulative nociceptive score 7 days post mCHI compared to control IgG. After additional sensitization with NTG, both acute sumatriptan treatment and chronic anti-CGRP mAb treatment produced antinociceptive effects on day 15 post mCHI (4 h post NTG).

Christensen et al. (109) examined the effect of a small molecule CGRP receptor antagonist, olcegepant, and a CGRP neutralizing antibody, ALD405, in order to better study the site of action of CGRP signaling antagonists in humans. Researchers hypothesize that the site of action is in the periphery. The NTG (10 mg/kg IP) mouse model was used in adult male C57BL/6 J mice. In this experiment, olcegepant (1 mg/kg IP or 0.45 μg/mouse ICV) and ALD405 (10 mg/kg IP or 10 μg/mouse ICV) were administered prior to measurement of cutaneous mechanical sensitivity. Olcegepant prevented NTG-induced allodynia when given systemically but not ICV, when compared to the vehicle + NTG groups. ALD405 also failed to prevent NTG induced allodynia when given ICV but produced antinociceptive effects when given systemically. Together, the data suggests that the site of action of CGRP inhibitors is outside of blood brain barrier and most likely peripheral consistent with most hypotheses on the efficacy of CGRP inhibitors in human migraineurs.

Ernstsen et al. (111) used the NTG mouse model of migraine to test the hypothesis that a combination of sumatriptan and the CGRP antagonist olcegepant would result in an additive effect. Prior to testing the drugs in combination, researchers tested dose-dependent relationships of sumatriptan (0.1, 0.3, and 0.6 mg/kg IP) and olcegepant (0.25, 0.50, and 1.0 mg/kg IP) on NTG-induced allodynia in male C57BL/6NATac mice. Both sumatriptan and olcegepant dose-dependently attenuated NTG-induced allodynia when given alone. The lowest dose of olcegepant, 0.25 mg/kg, did not significantly inhibit NTG provocation on any days. 0.50 mg/kg and 1.0 mg/kg olcegepant had significant effects on NTG provocation on day 1, day 3, day 5, and day 9. Next, researchers performed a combination study of the two drugs with a high and low dose. Following initial testing for low and high doses of sumatriptan, it was determined that all three doses reduced acute allodynia. For olcegepant, only 0.50 and 1.0 mg/kg had significant inhibitory effect on NTG-induced allodynia. For the following additive experiment, two combination groups were created, olcegepant 0.50 mg/kg + sumatriptan 0.1 mg/kg (combination 1) and olcegepant 0.50 mg/kg + sumatriptan 0.6 mg/kg (combination 2). When comparing the two combination groups to either the olcegepant or sumatriptan reference group, no significant difference was found on any days. These data strongly suggest the combination treatments had no greater effect on the NTG-induced allodynia compared to separate treatments.

Another study conducted by Greco et al. (113) explored the interplay between CGRP and other inflammatory mediators within the mechanisms of neuronal sensitization in an animal model of chronic migraine. Researchers used the CGRP antagonist olcegepant as a pharmacological probe in this study. Adult male Sprague Dawley rats received either acute or chronic systemic administration of NTG to produce cephalic and extracephalic hypersensitivity. After NTG treatment, animals underwent the orofacial formalin test to study the effect of olcegepant (1 and 2 mg/kg) on NTG-induced hyperalgesia. Acute NTG administration (10 mg/kg, IP) induced a hyperalgesic state that was detectable as an increase in nocifensive behavior (total face rubbing time) during Phase II of the orofacial formalin test. Of the doses tested, only 2 mg/kg of olcegepant significantly reduced NTG-induced nocifensive behavior, demonstrating a dose-dependent effect. Olcegepant (2 mg/kg) also significantly reduced nocifensive behavior following chronic NTG administration (5 mg/kg every other day for 10 days) compared to the NTG alone group. Researchers report that the changes in the CGRP pathway are paralleled by activation of the neuroinflammation cascade and demonstrate that CGRP receptor antagonism reduces the mediators of sensitization in the circuitry of migraines.

Wu et al. (114) sought to investigate the effect of CGRP receptor antagonist treatment on alleviating hyperalgesia and brain region activation in two rodent chronic migraine models. Male C57BL/6 J mice received repeated administration of either NTG or Levcromakalim (LEV) to model chronic migraine (138). Mice were injected with NTG or LEV every other day for 9 days. Wu et al. observed acute and basal mechanical hyperalgesia of these models, and then looked at the effect of CGRP receptor antagonist olcegepant. Olcegepant was administered IP 15 min prior to the LEV or NTG injection. Acute hyperalgesia was measured 2 h after NTG or LEV injection, whereas basal mechanical threshold (basal hyperalgesia) was measured prior to the VEH, NTG or LEV injection. Olcegepant (1 mg/kg) decreased NTG-induced acute, but not basal mechanical hyperalgesia of both the periorbital area (days 1, 5, and 9) and hindpaw (days 1, 3, 5, 7, and 9), compared to animals that received NTG alone. Similarly, olcegepant also alleviated acute and basal hyperalgesia in the LEV-induced chronic migraine model. They also evaluated the activation of different brain regions with c-Fos and NeuN staining. Overall, they report that olcegepant may alleviate mechanical hyperalgesia by attenuating brain activation. As seen above, numerous studies used NTG-induced models of migraine to evaluate the impact of CGRP-signaling inhibition. These data are generally consistent showing the CGRP inhibition reduces NTG-induced behavioral changes. The main exception to this is in the work from Christensen et al. (109) where ICV delivery of olcegepant failed to reduce NTG behavior highlighting the peripheral targeting of CGRP rather than the CNS.

### 4.2 Effect of CGRP inhibitors on migraine behavior in female rodents

Romero-Reyes et al. (115) investigated the role of CGRP in trigeminal orofacial pain of temporomandibular disorders by studying C57Bl/6 female mice using complete Freund’s adjuvant (CFA) to induce pain. While this is not a migraine model in itself, there is evidence of co-morbidity between temporomandibular disorders and migraine (139, 140). Therefore, we included this study in the present review. For behavioral assessment, mice were given an intramuscular injection of CFA (15 μL) into the right masseter muscle, then video recorded for assessment of spontaneous nociceptive-specific facial grooming patterns. The spontaneous grooming patterns include forepaw facial rubbing, chin/cheek rubbing, and hind paw face scratching performed by the mouse, as well as normal facial grooming. The CGRP receptor antagonist MK-8825 (70 mg/kg subcutaneous) had significant antinociceptive effects on the duration of forepaw face rubbing, chin and cheek rubbing, and hind paw scratching. The largest antinociceptive effect was demonstrated for forepaw face rubbing duration and the smallest effect was seen for the duration of the chin and cheek rubbing. These data suggest CGRP may be involved in temporomandibular disorder pathophysiology, similar to the CGRP effect in migraines.

Munro et al. (116) used female spontaneous trigeminal allodynia (STA) on the Sprague Dawley background. The STA rats display periorbital pain to cutaneous stimulation with von Frey filaments. In this study, researchers used the CGRP receptor antagonist olcegepant to measure CGRP antagonism on mechanical sensitivity of the face and paw. Analysis indicated that there was no significant effect of olcegepant (1 mg/kg, IP) on the hind paw withdrawal threshold, but there was a significant antinociceptive effect on the periorbital thresholds. No pronounced effect of olcegepant was observed in control female Sprague Dawley rats (i.e., no migraine model).

A study by Bree et al. (107) tested the ability of an anti-CGRP monoclonal antibody to ameliorate post-traumatic headache-like behavior in rats subjected to mild closed head injury (mCHI) pain behaviors in adult female Sprague Dawley rats. This was a follow-up study to their previous study, which was conducted in male Sprague Dawley rats (106). In this study, researchers report sex differences in the development of mechanical pain hypersensitivity following mCHI. While cephalic mechanical hypersensitivity in male rats subjected to mCHI resolved 14 days post-mCHI, mechanical hypersensitivity in female rats did not resolve until 29 days post-mCHI. Next, researchers used a low dose of NTG (100 ug/kg) to model post-trauma headache-like pain. They report that similar to males (106), female rats display mCHI-evoked prolonged latent cephalic sensitization to NTG. However, unlike male rats subjected to mCHI, female rats also display latent extracephalic sensitization to NTG.

Bree et al. (107) also investigated the effect of early and repeated administration of mouse anti-CGRP mAb on post-traumatic headache. Rats received mouse anti-CGRP mAb (30 mg/kg, IP) or its corresponding isotype IgG, immediately after the head injury and every 6 days subsequently up to 30 days. This group had previously reported that repeated administration of anti-CGRP mAb inhibited cephalic hypersensitivity and prolonged latent cephalic sensitation to NTG (106). Contrastingly, in the present study, similar administration of the anti-CGRP mAb in females failed to produce antinociceptive effects when compared with control IgG. Furthermore, anti-CGRP was also ineffective in inhibiting NTG-evoked hind paw hypersensitivity in female rats subjected to mCHI. Overall, the data outlined in this study compared to the male study suggest key sex differences regarding post-traumatic headache, including enhanced pain responses, an acute increase in anxiety-like behavior, and a decrease in efficacy to anti-CGRP mAb treatment in female rats.

Kopruszinski et al. (71) investigated the efficacy of current acute and preventative migraine medications to block priming and reverse umbellulone (UMB)-induced allodynia in female C57BL/6 J primed animals. Umbellulone, a monoterpene ketone that is an active constituent of the leaves of *Umbrellularia californica,* is thought to induce headache via the activation of transient receptor potential cation channel subfamily A, member 1 (TRPA1) channels in trigeminal nerve fibers and the release of CGRP (141). The medications used included propranolol (a beta blocker), sumatriptan, and the CGRP receptor antagonist olcegepant. Researchers used a “two-hit” hyperalgesic priming protocol that included consecutive episodes of restraint stress (first hit) and inhalation of UMB (second hit). To assess migraine-like pain, cutaneous allodynia was determined by responses to periorbital or hindpaw probing with von Frey filaments. In contrast to sumatriptan, olcegepant failed to attenuate UMB-induced periorbital hypersensitivity when compared to vehicle controls. However, when olcegepant was administered 30 min prior to UMB, olcegepant blocked the development of UMB-induced hypersensitivity. These data suggest that CGRP antagonists may be more effective when administered before the onset of a migraine than when the migraine is occurring.

Guo et al. (117) hypothesized that blocking both CGRP and PACAP signaling pathways may prevent migraine episodes and reduce migraine frequency and that recurring migraine episodes alter the strength of these pathways signaling in the trigeminal ganglion (TG) neurons. They used a NTG-induced migraine model with female CGRPα knockout mice and performed facial von Frey (117). Mechanical stimuli was measured at baseline and 2 days after each NTG injection. Results suggest that genetic deletion of CGRPα had a significant antinociceptive effect on von Frey thresholds compared to wild-type mice treated either acutely with NTG or repeatedly once per day. After repeated NTG administration, there was a significant increase in CGRP-R (*Calcrl*) neurons in mouse TG but not DRG, supporting its likely relevance to chronic migraine. Both NTG-induced behavioral sensitization and the increase in CGRP-R TG neurons were absent in CGRPα KO mice, indicating that both behavioral and cellular changes require the release of endogenous CGRP.

He et al. (104) sought to validate a behavioral assessment to capture the affective pain component in migraine using a mouse model of chronic NTG-induced migraine. In this study, the researchers used conditioned place preference (CPP) to observe ongoing spontaneous, affective, pain in adult female C57BL/6 mice. Mice received NTG (10 mg/kg, IP) every 2 days for a total of 4 injections (day 0, 2, 4, and 6) to develop migraine-like behaviors. NTG-treated and vehicle-treated mice received αCGRP _8–37_ systemically (i.v.) during the single-trial conditioning. When tested 24 h later, αCGRP_8-37_ (0.8 mg/kg, i.v.) produced pain relief-induced CPP in NTG-treated mice. NTG treated mice spent significantly more time in the chamber that was paired with αCGRP _8–37_ than that in the saline-paired chamber. This preference was not observed in vehicle-treated mice. Vehicle-treated mice did not show a preference for the αCGRP_8-37_ -paired or saline-paired chamber. Next, He et al. evaluated the chronicity of NTG-induced ongoing pain by extending the CPP protocol. He and collaborators waited 2 more days (72 h post last NTG exposure) before pairing mice with either saline or αCGRP_8-37_. Chronic NTG-pretreated mice displayed a significant preference for the αCGRP_8-37_ -paired chamber, while vehicle-pretreated mice spent a similar amount of time in saline-paired and CGRP_8-37_ -paired chambers.

### 4.3 Sex differences in the effect of CGRP inhibitors on migraine behavior

Christensen et al. (108) evaluated the role of CGRP receptor antagonism and antibody neutralization in two rodent models of migraine-like pain. For this study, experiments were conducted in male C57Bl/6 J mice or adult female STA rats. Christensen and collaborators observed the effect of olcegepant on acute hyperalgesia in NTG-treated and vehicle-treated male mice. NTG (10 mg/kg ip) or vehicle was administered 15 min after olcegepant (1 mg/kg, ip) or vehicle treatment on test days 1, 3, 5, 7, and 9. Researchers report that olcegepant prevented NTG-induced acute hyperalgesia on all test days (no effect on basal response/hyperalgesia). Additionally, the humanized monoclonal CGRP antibody, ALD405 (10 mg/kg, IP), also blocked NTG-induced acute and basal hyperalgesia in male mice. A separate experiment was performed in adult female STA rats. Both ALD405 (10 mg/kg) and olcegepant reversed cephalic hypersensitivity in von Frey of the periorbital area. ALD405 displayed a prolonged duration of action (up to 4 days) compared to olcegepant (up to 6 h). However, neither ALD405 nor olcegepant had an effect on hind paw hypersensitivity. Although, both male and female rodents were studied in this study, the different species used prevents a clear comparison between sexes.

Avona et al. (59) aimed to determine if two common migraine therapeutics, triptan and a CGRP monoclonal antibody, could decrease the response to nitric oxide (NO) donor in male and female stress-primed mice. Female and male ICR mice were subjected to restraint stress (via cylindrical rodent restrainer) for 2 h a day for 3 consecutive days, then von Frey was conducted 24 h after the final stress session. The authors report that repeated restraint stress (RS) induced significant facial hypersensitivity in both males and females. Next, Avona et al. sought to determine whether RS caused priming to the NO-donor. Mice were injected with 0.1 mg/kg sodium nitroprusside (SNP) following a return to baseline. Both male and female SNP-treated mice displayed significant facial hypersensitivity 1- and 3 h following SNP injection. To determine the possible role of CGRP in stress-induced hypersensitivity, mice received ALD405 (10 mg/kg, IP) or an isotype control 24 h following the return to baseline in RS mice. Mice then received SNP treatment 24 h after ALD405 or isotype control treatment. ALD405 significantly blocked the effects of SNP in both females and males. However, sex differences were observed in the antinociceptive effect of ALD405 in stress-primed mice. While ALD405 significantly blocked the effects of SNP in females from 1–72 h following SNP injection, ALD405 only attenuated mechanical sensitivity at 48 h following SNP injection in male mice. These data suggest that CGRP has a sexually dimorphic role in stress-induced priming to NO donor application.

Christensen et al. (110) investigated the signaling pathways involved in mouse models of provoked migraine. This study utilized three different mouse models of provoked migraine (i.e., NTG, cilostazol, and LEV and measured hypersensitivity to tactile stimulation. Cilostazol is a phosphodiesterase 3 (PDE_3_) inhibitor that causes cyclic adenosine monophosphate (cAMP) accumulation, inducing migraine-like attacks in both healthy subjects and migraineurs without aura (142, 143). While levcromakalim, an ATP-sensitive potassium (K_ATP_) channel opener, known to induce an acute rapid onset of migraine-like behavior likely through dilation of cranial arteries (144). Specific knockout mice and chemical inhibitors were used to understand the signaling pathways that were involved in each model. Although both male and female mice were used in this study, data from both sexes were pooled. The researchers studied the effect of the CGRP monoclonal antibody ALD405 and the CGRP antagonist olcegepant (1 mg/kg IP) on cilostazol- and levcromakalim-induced hypersensitivity. They found that ALD405 and olcegepant both attenuated cilostazol- and levcromakalim-induced hypersensitivity. While ALD405 fully inhibited the mechanical hypersensitivity, olcegepant only partially attenuated cilostazol- and levcromakalim-induced hypersensitivity. They also looked at the effect of levcromakalim on mechanical hypersensitivity in Ramp1 KO mice. They found that Ramp1 KO mice were resistant to the effect of levcromakalim when compared to wildtype controls. The authors conclude that the three mouse models of provoked migraine all involve CGRP signaling.

Ernstsen and collaborators sought to understand the differences in CGRP- and PACAP-pathways in migraine-like pain (112). Male or female *RAMP1* KO mice and *RAMP1* WT mice (littermate controls) were treated with NTG (10 mg/kg, IP) or vehicle. Cephalic mechanical hypersensitivity was observed on days 1, 3, 5, 7, and 9. NTG induced a significant increase in mechanical hypersensitivity in *RAMP1* WT mice, acutely (2 h after administration). However, this mechanical sensitivity was not observed in *RAMP1* KO mice. *RAMP1* KO mice were “protected” from the effects of NTG, including acute diarrhea. The authors report that while no apparent sex differences were noticeable in the experiments, a limitation of the study was that the experiments were not designed to power sex differences.

Viero et al. characterized nociception and anxiety-related symptoms after induction of sound stress in male and female adult C57BL/6 mice (58). Researchers note that patients with migraine have higher levels of plasma inflammatory cytokines and CGRP, and although stress mediated by unpredictable sound is already used as a model of painful sensitization, migraine-like behaviors and sex-linked differences have not been evaluated. C57BL/6 female and male mice were treated with BIBN4096BS (olcegepant) (IP 100 mg/kg). The behavioral tests were conducted from least to most stress-inducing starting with grimace, hind paw mechanical allodynia, periorbital mechanical allodynia (PMA), and then the open field test. Results showed that seven days post-stress nociception behaviors were consistently abolished by olcegepant in both female and male mice.

Chou et al. (118) investigated cell-types within the central amygdala (CeA) and their contribution to migraine chronification. Male and female C57BL/6 J mice were used to measure mechanical sensitivity, CGRP expression in trigeminal ganglion, and responsiveness to CGRP_8-37_ following NTG injection. The data from both male and females was pooled together in this study. An antinociceptive effect was defined as an increase in mechanical threshold. CGRP_8-37_ was applied into bilateral CeA two hours after NTG administration every other day for nine days. CGRP_8-37_ attenuated NTG-induced mechanical hyperalgesia compared to vehicle controls. Additionally, the expression level of pERK and co-expressed PKC-*δ* positive neurons decreased after CGRP_8-37_ injection. Researchers concluded CeA PKC-δ positive neurons innervated by CGRP positive neurons may contribute to chronification of migraine and note this may contribute to the increased CGRP release from the parabrachial nucleus. The researchers did not note any significant sex differences within their experiment.

Navratilova et al. (119) explored possible sex differences in the effects of CGRP receptor inhibition in a mouse model of mild traumatic brain injury (mTBI)-induced PTH in male or female mice. For this study, lightly anesthetized mice received a weight drop onto a closed-skull, followed by a rotational flip, to model mTBI. Following mTBI induction, pain-like behavior was measured for a time course of up to 14 or 28 days in response to olcegepant (3 mg/kg 2 h after mTBI or sham and on days 3, 6, 9, and 12 post-induction) or vehicle administration. Initial pain behaviors (periorbital allodynia, hindpaw allodynia, and thermal sensitivity) were interpreted as modeling acute post-traumatic headache (APTH), and were resolved by day 14 post-mTBI. Persistent post-traumatic headache (PPTH), was interpreted as long-lasting allodynia induced by a second subthreshold trigger – bright light stress (BLS), over a 6 h time course period. While olcegepant reduced periorbital, hindpaw, and thermal allodynia in both male and female mice, there was greater efficacy observed in females, compared to males. In the case of PPTH, early and repeated injections of olcegepant prevented BLS-induced periorbital allodynia, hindpaw allodynia and thermal allodynia in both male and female mice up to day 28 (16 days after the last olcegepant treatment). Similarly to APTH, the antinociceptive effects of olcegepant was significantly greater in female mice compared to males with BLS-induced PPTH. The sex differences in the magnitude of antinociception are similar to what was observed in the study by Avona et al. (59) ALD405 produced antinociceptive effects in both male and female mice, but ALD405 was more effective in blocking responses to SNP following stress in female mice than in male mice. Lastly, this study sought to study the effect of CGRP inhibition to prevent BLS-induced allodynia in male and female mice. Male or female mice were given a single mTBI (or sham) treatment. On day 14, and again on day 28, mice were treated with olcegepant (3 mg/kg) 1 h before BLS exposure. Olcegepant treatment before BLS showed minimal-to-no efficacy in preventing the development of BLS-induced periorbital and hindpaw allodynia in either female or male mice. These data suggest that early and sustained CGRP-targeting interventions following mTBI could be beneficial in preventing and managing traumatic-induced headaches, with greater efficacy observed in females.

Taken together, when comparing the effects of CGRP antagonism, there are general observations of antinociception and attenuation of photophobia and anxiety-like behavior in both male and female rodents. In some studies, sex differences were observed in the efficacy of anti-CGRP interventions. The antinociceptive effects of CGRP inhibitors are similar to those observed in migraine patients and non-headache rodent pain models. CGRP antagonists – CGRP_8-37_, BIBN4096BS (olcegepant), and MK-8825 have produced antinociceptive effects in rodent models of somatic and visceral pain (145–148). Collectively, data from these studies largely supports targeting CGRP or its receptor for attenuating migraine symptoms.

## 5 Discussion

After puberty, migraine is predominantly reported in women, who are three times more likely to suffer from migraines than men (16, 17). While animal studies have shed some light on sex differences in migraine, this sex disparity remains poorly understood (98, 125, 149, 150). The initial discovery of CGRP within the trigeminovascular system was reported in the 1980s, representing a significant milestone in the advancement of migraine research (151, 152). Since then, few studies have focused on sex-differential expression levels of CGRP and CGRP-related genes and receptors under naïve conditions and/or in rodent models that mimic migraine. In naïve conditions, RAMP1 mRNA levels have been shown to be significantly higher in female trigeminal ganglia than in males (153). Contrastingly, baseline mRNA levels of the genes encoding CGRP receptor components (i.e., RAMP1, CLR, and RCP) have also been reported to be higher in the trigeminal ganglia of males than females, but no baseline sex differences in CGRP-encoding mRNA (154). In an inflammatory soup model of migraine, there was an increase in expression of mRNAs encoding CGRP, RAMP1, RCP, and CLR in the trigeminal ganglia (154) of males; however, only CLR and RCP levels were increased in females. Sex differences in the effect of CGRP have been shown in rodent pain models outside of migraine (155).

The primary purpose of this review was to outline current preclinical literature investigating the effect of CGRP and CGRP antagonism on migraine-like behavior and evaluate sex differences in the context of CGRP in migraine-associated behaviors in rodents. Stress, sensory stimuli, sleep schedule, diet, and gender are all factors that contribute to migraine pain (156–159). Unlike human studies, many of these factors can be controlled in animal studies. Additionally, both sex and gender play instrumental roles in the risk, pathophysiology, presentation of symptoms associated with migraine, and care received (160). Few clinical studies account for gender in study design and analysis (161). Animal models of migraine allow researchers to focus on biological sex differences, without gender as an additional variable. Therefore, animal studies are likely critical to understanding the role of CGRP inhibitors in migraine-associated behavior. To our knowledge, this is the first review to look at the effect of CGRP and CGRP antagonists on migraine-like pain in rodents stratified by sex.

In this review, we included 35 manuscripts with goals to evaluate potential sex differences in the effects of (1) CGRP and (2) CGRP inhibition on migraine-like pain in rodents. For the first goal, CGRP appears to have a general pronociceptive effect in behavioral studies that use both male and female rodents with a more consistent and severe phenotype in females (Figures 2, 3). Findings from male and female subjects were either pooled or not statistically powered to detect sex differences in four of the 10 studies (40%) (93–95, 100). Six studies included both sexes and analyzed the data by sex. Of the six studies that were analyzed by sex, four studies (~67%) reported significant sex differences (61, 98, 99, 101).

These findings are consistent with clinical reports of characteristic symptoms associated with migraine (e.g., phonophobia, photophobia, allodynia, and anxiety) being more frequent among women than men (162–165). Interestingly, a female-specific role for CGRP has also been observed in non-headache rodent pain models. In a model of neuropathic pain, an increase in CGRP, CLR, and RAMP1 mRNA expression was observed in the central amygdala of female rats compared to males at the chronic phase while males showed an increase of CGRP mRNA expression at the acute phase of neuropathic pain (166). The exact reasons for the enhanced CGRP-mediated pain response in female rodents during the chronic phase are not fully understood; however, preclinical studies have consistently demonstrated a female selectivity of prolactin which may be involved (167). Prolactin has been shown to selectively sensitize female nociceptors, increase the release of CGRP, and when applied to the dura, prolactin has been shown to produce migraine-like pain in female, but not male mice (125, 168).

Despite the substantive potential for sex differences with CGRP, of the six studies that analyzed by sex, two studies (~ 33%) did not observe significant sex differences and reported CGRP to have similar effects on migraine-like behavior in males and females (96, 97). In female rodents, the estrous cycle plays a crucial role in some types of behavior. Female mice in proestrus have demonstrated a greater sensitivity to nociceptive stimuli than other stages of the estrous cycle (169). Animal studies have shown that activation of the CGRP system varies at different stages of the estrous cycle (170). It is possible that CGRP effects in migraine models could be influenced by the estrous cycle. On the other hand, estrus cycle monitoring is typically invasive, making estrus monitoring a non-trivial addition to the modeling of a disease with a significant stress component. In the future, consideration of the estrus stage may provide additional nuance to understanding the role of CGRP antagonism in rodent models of migraine. The pronociceptive effects induced by CGRP in these studies are consistent with clinical studies that have examined CGRP in both male and female patients with migraines and healthy controls. Intravenous infusions of CGRP have been shown to induce migraine-like headaches and trigger migraine like-attacks in numerous human studies (171–173). A study by Kamm et al. (174) found elevated CGRP concentrations in tear fluid in chronic migraine patients compared to healthy controls. Additionally, the CGRP concentrations of unmedicated ictal migraine patients were more elevated than medicated ictal migraine patients (174). Elevated CGRP levels have also been seen in patients with somatic, non-headache pain (175, 176).

The second goal of the review was to study the effect of CGRP inhibitors on pain-like behavior in animal studies. FDA-approved antibodies and antagonists have been shown to attenuate migraine-related pain in animal subjects (177, 178) (Table 1). CGRP antagonists, monoclonal antibodies, and transgenic animals (Ramp1 KO) all appear to have a primarily antinociceptive effect on behavioral assays in studies using only male or female rodents. In single-sex studies (i.e., only males or only females), antinociceptive effects were seen in all of the studies. However, Bree et al., noticed sex differences of anti-CGRP mAb treatment in male (106) and female (107) NTG-treated rats in two separate studies. Of the 22 papers that studied the effect of CGRP inhibition on migraine-like behavior, 7 studies (~ 32%) compared males and females in a single study. Of those 7 studies, 4 studies either included pooled data from both males and females (110, 118), did not have adequate statistical power to detect sex differences (111), or used male and female animals of different strains (108). Three studies, included data stratified by sex. Of these three studies, 1 study (~ 33%) by Viero et al. (58) showed similar antinociceptive effects in male and females. Two of the three studies (~ 67%) observed sex differences in CGRP-R inhibition (59, 119). The antinociceptive effects of CGRP inhibitors are similar to those observed in migraine patients and non-headache rodent pain models. CGRP antagonists - CGRP_8-37_, BIBN4096BS (olcegepant), and MK-8825 have produced antinociceptive effects in rodent models of somatic and visceral pain (145–147, 179). The sex differences seen by Avona et al. (50) are consistent with the sex differences in the efficacy of CGRP-targeting therapies in male and female patients. This review adds to the body of evidence supporting CGRP antagonists and monoclonal antibodies as effective compounds for migraine relief and prevention.

There is clearly a lack of published data on sex differences related to CGRP inhibition. These data also highlight the need for future studies to directly compare the effects of antagonists in male versus female rodents. Such studies, accompanied by molecular experiments and analyses, will help identify the mechanisms driving sex differences in migraine development, maintenance, and treatment. It is worth noting that evidence of sex differences in CGRP inhibition has been observed in clinical trials (148). The CGRP receptor antagonists - ubrogepant and rimegepant have been reported to be less effective for migraine relief in men compared to women (43, 45). However, it is unknown how much other factors, such as weight and gender, contribute to this reported sex difference.

Although CGRP monoclonal antibodies and antagonists have been shown to decrease migraine-associated behaviors, they are not the first choice therapy for migraine prevention (180). This is primarily due to the fact that most health insurance providers still do not cover the costs of CGRP monoclonal antibodies and antagonists (181). Knowledge gaps exist regarding the long-term effects of inhibiting the CGRP pathway, drug-to-drug interactions of CGRP antibodies and other medications, and potential sex differences that may exist. This review sought to compare the influence of CGRP on migraine-associated behaviors in rodent migraine models. Despite limitations, the existing mouse and rat literature suggests that antagonists and drugs targeting CGRP may be effective in both males and females, with potential for greater efficacy in females. This small but potentially meaningful difference between male and female rodents provides motivation to use rodent models to explore the mechanisms of sex differences in migraine to develop novel targets for migraine therapy in pain.

## Footnotes

^1^ https://pubmed.ncbi.nlm.nih.gov/
