# Supplementary material for: Sex differences in the effects of calcitonin gene-related peptide signaling on migraine-like behavior in animal models: a narrative review
Source: Front Neurol. 2025 Jul 10;16:1603758. doi: 10.3389/fneur.2025.1603758 (PMC12288688; doi:10.3389/fneur.2025.1603758)
Supplement: Supplementary file 1 [file Table_1.docx]

**Supplemental Table 1: Summary of all Behavior for Studies Evaluating CGRP Direct Effects on Migraine-like Behavior**

| **Source** | **Pain Assay/Test** | **Migraine**  **Model** | **Animal** | **Sex** | **Figure in Paper** | **Treatment** | **Main Effect** |
| --- | --- | --- | --- | --- | --- | --- | --- |
| Huang et al. 2016 | Nocifensive behavior |  | Swiss-Webster mice | M | Fig 7C | Dura CGRP (100 mM) | No effect |
|  | Nocifensive behavior |  | Swiss-Webster mice | M | Fig 7C | Dura CGRP (1 mM) | No effect |
| Cornelison et al. 2016 | von Frey  (nocifensive head withdrawal) |  | Sprague-Dawley | M | Fig 1A | Intracisternal CGRP (1 mM) | Pronociception |
| Yao et al. 2017 | Climbing hutch behavior |  | Sprague-Dawley | M | Fig 1 | Epidural CGRP (1.5 mg) | Pronociception |
|  | Climbing hutch behavior |  | Sprague-Dawley | M | Fig 1 | Epidural CGRP (3 mg) | Pronociception |
|  | Climbing hutch behavior |  | Sprague-Dawley | M | Fig 1 | Epidural CGRP (6 mg) | Pronociception |
|  | Climbing hutch behavior |  | Sprague-Dawley | M | Fig 1 | Epidural CGRP (9 mg) | Pronociception |
|  | Facial grooming |  | Sprague-Dawley | M | Fig 2C | Epidural CGRP (1.5 mg) | Decreased facial grooming |
|  | Facial grooming |  | Sprague-Dawley | M | Fig 2D | Epidural CGRP (3 mg) | Decreased facial grooming |
|  | Facial grooming |  | Sprague-Dawley | M | Fig 2E | Epidural CGRP (6 mg) | Decreased facial grooming |
|  | Facial grooming |  | Sprague-Dawley | M | Fig 2F | Epidural CGRP (9 mg) | Decreased facial grooming |
| De Logu et al. 2019 | Nociceptive behavior |  | C57BL/6J mice | M | Fig 1B | Subcutaneous CGRP  (15 nmol/p.orb.) | No effect |
|  | von Frey  (periorbital) |  | C57BL/6J mice | M | Fig 1C | Subcutaneous CGRP  (0.15 nmol/p.orb.) | Pronociception |
|  | von Frey  (periorbital) |  | C57BL/6J mice | M | Fig 1C | Subcutaneous CGRP  (1.5 nmol/p.orb.) | Pronociception |
|  | von Frey  (periorbital) |  | C57BL/6J mice | M | Fig 1C | Subcutaneous CGRP  (15 nmol/p.orb.) | Pronociception |
| Recober et al. 2009 | Time in light (per 300 sec) |  | nestin/hRAMP1 mice | F | Fig 1B | Elevated RAMP1 expression | Pronociception |
|  | Time in light (per 300 sec) |  | nestin/hRAMP1 mice | M | Fig 1B | Elevated RAMP1  expression | Pronociception |
| Marquez de Prado et al. 2009 | von Frey  (hindpaw) |  | Control mice  (littermates) | M/F | Fig 3A | Intrathecal CGRP  (1 nmol) | No effect |
|  | von Frey  (hindpaw) |  | Control mice  (littermates) | M/F | Fig 3A | Intrathecal CGRP  (5 nmol) | Pronociception |
|  | von Frey  (hindpaw) |  | nestin/hRAMP1  mice | M/F | Fig 3B | Intrathecal CGRP  (1 nmol) | Pronociception |
| Recober et al. 2010 | Time in light  (1^st^ and 2^nd^ 300 sec) |  | Control mice  (littermates) | M/F | Fig 2 | Central CGRP  (0.5 nmol, ICV) | No effect |
|  | Time in light  (1^st^ and 2^nd^ 300 sec) |  | nestin/hRAMP1  mice | M/F | Fig 2 | Central CGRP  (0.5 nmol, ICV) | Light aversion |
|  | Rearing (#/sec) – Dark zone  (1^st^ 300 sec) |  | Control mice  (littermates) | M/F | Fig 5 | Central CGRP  (0.5 nmol, ICV) | No effect |
|  | Rearing (#/sec) – Dark zone  (2^nd^ 300 sec) |  | Control mice  (littermates) | M/F | Fig 5 | Central CGRP  (0.5 nmol, ICV) | Reduced motility  (reduced rearing) |
|  | Rearing (#/sec) – Light zone  (1^st^ 300 sec) |  | nestin/hRAMP1  mice | M/F | Fig 5 | Central CGRP  (0.5 nmol, ICV) | No effect |
|  | Rearing (#/sec) – Light zone  (2^nd^ 300 sec) |  | nestin/hRAMP1  mice | M/F | Fig 5 | Central CGRP  (0.5 nmol, ICV) | No effect |
| Mason et al. 2017 | Time in light (sec) |  | CD1 mice | M/F | Fig 1A | Peripheral CGRP  (0.1 mg/kg, IP) | Light aversion |
|  | Time in light (sec) |  | C57BL/6J mice | M/F | Fig 1B | Peripheral CGRP  (0.1 mg/kg, IP) | Light aversion |
|  | Resting time (%)  Dark zone |  | CD1 mice | M/F | Fig 2A | Peripheral CGRP  (0.1 mg/kg, IP) | Reduced motility  (Increased resting) |
|  | Resting time (%)  Light zone |  | CD1 mice | M/F | Fig 2A | Peripheral CGRP  (0.1 mg/kg, IP) | No effect |
|  | Resting time (%)  Dark zone |  | C57BL/6J mice | M/F | Fig 2A | Peripheral CGRP  (0.1 mg/kg, IP) | Reduced motility  (Increased resting) |
|  | Resting time (%)  Light zone |  | C57BL/6J mice | M/F | Fig 2A | Peripheral CGRP  (0.1 mg/kg, IP) | No effect |
|  | Vertical Beam Breaks  (Rearing – Dark zone) |  | CD1 mice | M/F | Fig 2B | Peripheral CGRP  (0.1 mg/kg, IP) | Reduced motility  (Reduced resting) |
|  | Vertical Beam Breaks  (Rearing – Light zone) |  | CD1 mice | M/F | Fig 2B | Peripheral CGRP  (0.1 mg/kg, IP) | No significant effect |
|  | Vertical Beam Breaks  (Rearing – Dark zone) |  | C57BL/6J mice | M/F | Fig 2B | Peripheral CGRP  (0.1 mg/kg, IP) | Reduced motility  (Reduced rearing) |
|  | Vertical Beam Breaks  (Rearing – Light zone) |  | C57BL/6J mice | M/F | Fig 2B | Peripheral CGRP  (0.1 mg/kg, IP) | Reduced motility  (Reduced rearing) |
|  | Time in light (sec) |  | C57BL/6J mice | M/F | Fig 3A | Central CGRP  (2 μg, ICV) | Light aversion |
|  | Resting time (%)  Dark zone |  | C57BL/6J mice | M/F | Fig 3B | Central CGRP  (2 μg, ICV) | Reduced motility  (Increased resting) |
|  | Resting time (%)  Light zone |  | C57BL/6J mice | M/F | Fig 3B | Central CGRP  (2 μg, ICV) | No effect |
| Rea et al. 2018 | Mean grimace score |  | CD1 mice | F | Suppl. Fig 3 | Peripheral CGRP  (0.1 mg/kg) | Pronociception |
|  | Grimace score (Tx1) |  | CD1 mice | F | Fig 2C | Peripheral CGRP  (0.1 mg/kg) | Pronociception |
|  | Mean grimace score |  | CD1 mice | M | Suppl.  Fig 3 | Peripheral CGRP  (0.1 mg/kg) | Pronociception |
|  | Grimace score (Tx1) |  | CD1 mice | M | Fig 2B | Peripheral CGRP  (0.1 mg/kg) | Pronociception |
| Avona et al. 2019 | von Frey  (facial hypersensitivity) |  | Sprague-Dawley | F | Fig 1A | Dural CGRP  (3.8 mg) | Pronociception |
|  | von Frey  (facial hypersensitivity) |  | Sprague-Dawley | M | Fig 1A | Dural CGRP  (3.8 mg) | No significant effect |
|  | von Frey  (facial hypersensitivity) |  | Sprague-Dawley | F | Fig 2 | Dural CGRP  (1 pg) | Pronociception |
|  | von Frey  (facial hypersensitivity) | IL-6 priming | Sprague-Dawley | F | Fig 3A | Dural CGRP  (0.1 pg) | Pronociception |
|  | von Frey  (facial hypersensitivity) | IL-6 priming | Sprague-Dawley | M | Fig 3B | Dural CGRP  (0.1 pg) | No effect |
|  | von Frey  (facial hypersensitivity) |  | ICR mice | F | Fig 5A | Dural CGRP  (1 pg) | Pronociception |
|  | von Frey  (facial hypersensitivity) |  | ICR mice | M | Fig 5A | Dural CGRP  (1 pg) | No effect |
|  | Grimace |  | ICR mice | F | Fig 5B | Dural CGRP  (1 pg) | Pronociception |
|  | Grimace |  | ICR mice | M | Fig 5B | Dural CGRP  (1 pg) | No effect |
|  | von Frey  (hindpaw hypersensitivity) |  | Sprague-Dawley | F | Fig 7D | Intraplantar CGRP  (1 pg) | Pronociception |
|  | von Frey  (hindpaw hypersensitivity) |  | Sprague-Dawley | M | Fig 7B | Intraplantar CGRP  (1 pg) | No effect |
| Araya et al. 2020 | von Frey (periorbital) |  | Wistar rats | M | Fig 1B | Intraganglionar CGRP  (0.1 nmol/10 mL) | Pronociception |
|  | von Frey (periorbital) |  | Wistar rats | F | Fig 1C | Intraganglionar CGRP  (0.1 nmol/10 mL) | Pronociception |
|  | von Frey  (after aversive light) |  | Wistar rats | M | Fig 4B | Intraganglionar CGRP  (0.1 nmol/10 mL) | Pronociception |
|  | von Frey  (after aversive light) |  | Wistar rats | F | Fig 4C | Intraganglionar CGRP  (0.1 nmol/10 mL) | Pronociception |
| Wattiez et al. 2021 | Distance traveled (10 AM) |  | CD1 mice | M/F | Fig 1B  (right) | Peripheral CGRP  (0.1 mg/kg IP) | Decrease in distance traveled |
|  | Distance traveled (2 PM) |  | CD1 mice | M/F | Fig 1C  (right) | Peripheral CGRP  (0.1 mg/kg IP) | Decrease in distance traveled |
|  | Distance traveled (8 PM) |  | CD1 mice | M/F | Fig 1D  (right) | Peripheral CGRP  (0.1 mg/kg IP) | Decrease in distance traveled |
|  | Wheel revolutions (1^st^ hr)  (10 AM) |  | CD1 mice | M/F | Fig 2C | Peripheral CGRP  (0.1 mg/kg IP) | Decrease in wheel revolutions |
|  | Wheel revolutions (2^nd^ hr)  (10 AM) |  | CD1 mice | M/F | Fig 2C | Peripheral CGRP  (0.1 mg/kg IP) | No effect |
|  | Wheel revolutions (1^st^ hr)  (8 PM) |  | CD1 mice | M/F | Fig 2D | Peripheral CGRP  (0.1 mg/kg IP) | Decrease in wheel revolutions |
|  | Wheel revolutions (2^nd^ hr)  (8 PM) |  | CD1 mice | M/F | Fig 2D | Peripheral CGRP  (0.1 mg/kg IP) | No effect |
|  | Time in light (test 1)  10 AM |  | CD1 mice | M/F | Fig 3B  (right) | Peripheral CGRP  (0.1 mg/kg IP) | Decreased time in light |
|  | Time in light (test 2)  8 PM |  | CD1 mice | M/F | Fig 3B  (right) | Peripheral CGRP  (0.1 mg/kg IP) | Decreased time in light |
| Wang et al. 2022 | Time in light |  | C57BL/6J mice | M | Fig 1 | Central CGRP  (1 μg/200 nl) | Decreased time in light |
|  | Time in light |  | C57BL/6J mice | F | Fig 1 | Central CGRP  (1 μg/200 nl) | Decreased time in light |
|  | Mobility – Dark  (Resting time %) |  | C57BL/6J mice | M | Fig 2  A-B | Central CGRP  (1 μg/200 nl) | Increased resting % |
|  | Mobility – Dark  (Resting time %) |  | C57BL/6J mice | F | Fig 2  A-B | Central CGRP  (1 μg/200 nl) | Increased resting % |
|  | Mobility – Dark  Vertical beam breaks |  | C57BL/6J mice | M | Fig 2  C-D | Central CGRP  (1 μg/200 nl) | Trend – decreased vertical beam breaks |
|  | Mobility – Dark  Vertical beam breaks |  | C57BL/6J mice | F | Fig 2  C-D | Central CGRP  (1 μg/200 nl) | Trend – decreased vertical beam breaks |
|  | Mobility  # of transitions |  | C57BL/6J mice | M | Fig 2  E-F | Central CGRP  (1 μg/200 nl) | Decrease in # of transitions |
|  | Mobility  # of transitions |  | C57BL/6J mice | F | Fig 2  E-F | Central CGRP  (1 μg/200 nl) | Decrease in # of transitions |
|  | von Frey – hindpaw  (contralateral to inj.) |  | C57BL/6J mice | M | Fig 5A | Central CGRP  (1 μg/200 nl) | Pronociception |
|  | von Frey – hindpaw  (contralateral to inj.) |  | C57BL/6J mice | F | Fig 5A | Central CGRP  (1 μg/200 nl) | Pronociception |
|  | von Frey – hindpaw  (ipsilateral to inj.) |  | C57BL/6J mice | M | Fig 5C | Central CGRP  (1 μg/200 nl) | Pronociception |
|  | von Frey – hindpaw  (ipsilateral to inj.) |  | C57BL/6J mice | F | Fig 5C | Central CGRP  (1 μg/200 nl) | Pronociception |
|  | Nociceptive squinting |  | C57BL/6J mice | M | Fig 6C | Central CGRP  (1 μg/200 nl) | No effect |
|  | Nociceptive squinting |  | C57BL/6J mice | F | Fig 6B | Central CGRP  (1 μg/200 nl) | Pronociception |
| Guzman et al. 2025 | von Frey - periorbital |  | C57BL/6J mice | F | Fig 2A | Supradural CGRP  (1 pg/5 μl) | Pronociception |
|  | von Frey - periorbital |  | C57BL/6J mice | M | Fig 2B | Supradural CGRP  (1 pg/5 μl) | No effect |
|  | von Frey - hindpaw |  | C57BL/6J mice | F | Supp.  Fig 1A | Supradural CGRP  (1 pg/5 μl) | Pronociception |
|  | von Frey - hindpaw |  | C57BL/6J mice | M | Supp.  Fig 1B | Supradural CGRP  (1 pg/5 μl) | No effect |
|  | von Frey - periorbital | Chronic  NTG | C57BL/6J mice | F | Fig 3E | Supradural CGRP  (0.1 pg/5 μl) | Pronociception |
|  | von Frey - periorbital | Chronic  NTG | C57BL/6J mice | M | Fig 3F | Supradural CGRP  (1 pg/5 μl) | Pronociception |
|  | von Frey - hindpaw | Chronic  NTG | C57BL/6J mice | F | Supp.  Fig 2E | Supradural CGRP  (0.1 pg/5 μl) | Pronociception |
|  | von Frey - hindpaw | Chronic  NTG | C57BL/6J mice | M | Supp.  Fig 2F | Supradural CGRP  (1 pg/5 μl) | Pronociception |
|  | von Frey - periorbital | MOH | C57BL/6J mice | F | Fig 6E | Supradural CGRP  (0.1 pg/5 μl) | Pronociception |
|  | von Frey - periorbital | MOH | C57BL/6J mice | M | Fig 6F | Supradural CGRP  (1 pg/5 μl) | Pronociception |
|  | von Frey - hindpaw | MOH | C57BL/6J mice | F | Supp.  Fig 3E | Supradural CGRP  (0.1 pg/5 μl) | Pronociception |
|  | von Frey - hindpaw | MOH | C57BL/6J mice | M | Supp.  Fig 3F | Supradural CGRP  (1 pg/5 μl) | Pronociception |

**Supplemental Table 2: Summary of all Behavior for Studies Evaluating CGRP Receptor Inhibition on Migraine-like Behavior**

| **Source** | **Pain Assay/Test** | **Migraine**  **Model** | **Animal** | **Sex** | **Figure in Paper** | **Treatment** | **Main Effect** |
| --- | --- | --- | --- | --- | --- | --- | --- |
| Martino et al. 2008 | Ultrasound vocalizations | LPS | Sprague-Dawley | M | Fig 8A | hCGRP_8-37_ | Antinociception |
|  | Ultrasound vocalizations | LPS | Sprague-Dawley | M | Fig 8B | BIBN4096BS | Antinociception |
| Greco et al. 2014 | Tail flick | NTG | Sprague-Dawley | M | Fig 1C | MK8825 | Antinociception |
|  | Formalin test (phase I) | NTG | Sprague-Dawley | M | Fig 2C | MK8825 | No effect |
|  | Formalin test (phase II) | NTG | Sprague-Dawley | M | Fig 2C | MK8825 | Antinociception |
| Huang et al. 2016 | Nocifensive behavior | ISCap | Swiss-Webster mice | M | Fig 7A | CGRP_8-37_ | Antinociception |
|  | Hindpaw scratching | ISCap | Swiss-Webster mice | M | Fig 7B | CGRP_8-37_ | Antinociception |
|  | Forepaw wiping | ISCap | Swiss-Webster mice | M | Fig 7B | CGRP_8-37_ | Antinociception |
|  | Nocifensive behavior | ISCap | CDI mice | M | Fig 9G | CGRP_8-37_ | Antinociception |
| He et al. 2021 | CPP | NTG | C57BL/6J mice | F | Fig 1  B-C | CGRP_8-37_ | Antinociception-induced CPP |
|  | CPP | Chronic  NTG | C57BL/6J mice | F | Fig 1  B-C | CGRP_8-37_ | Antinociception-induced CPP |
| Avona et al. 2020 | von Frey  facial hypersensitivity | RS/SNP | ICR | M | Fig 4A | ALD405 | Antinociception* |
|  |  | RS/SNP | ICR | F | Fig 4B | ALD405 | Antinociception |
| Daiutolo et al. 2016 | von Frey (ipsilateral) | CCI | C57BL/6J mice | M | Fig 2 | MK8825 | Antinociception |
|  | von Frey (contralateral) | CCI | C57BL/6J mice | M | Fig 2 | MK8825 | No effect |
|  | Light-dark (% Time in light) | CCI | C57BL/6J mice | M | Fig 3B | MK8825 | Improved CCI-induced photophobia |
|  | Light-dark (transitions) | CCI | C57BL/6J mice | M | Fig 3C | MK8825 | No effect |
|  | Light-dark (rears) | CCI | C57BL/6J mice | M | Fig 3D | MK8825 | Improved CCI-induced photophobia |
| Bree et al. 2018 | Facial von Frey | Low dose  GTN | Sprague-Dawley | M | Fig 3C | Anti-CGRP mAb | Antinociception |
|  | Cumulative nociceptive score | Low dose  GTN | Sprague-Dawley | M | Fig 3D | Anti-CGRP mAb | Antinociception |
|  | von Frey  (decrease in threshold %) | Low dose  GTN | Sprague-Dawley | M | Fig 7C | Anti-CGRP mAb | Antinociception |
|  | Nociceptive score  (increase in score %) | Low dose  GTN | Sprague-Dawley | M | Fig 7D | Anti-CGRP mAb | Antinociception |
| Bree et al. 2020 | von Frey  (pericranial/cephalic) | mCHI | Sprague-Dawley | F | Fig 6B | Anti-CGRP mAb | No effect |
|  | Cephalic von Frey  (decrease in threshold %) | GTN/mCHI | Sprague-Dawley | F | Fig 7B | Anti-CGRP mAb | Antinociception |
|  | Increase in pain score % | GTN/mCHI | Sprague-Dawley | F | Fig 7C | Anti-CGRP mAb | No significant effect |
|  | Extracephalic von Frey  (decrease in threshold %) | GTN/mCHI | Sprague-Dawley | F | Fig 7D | Anti-CGRP mAb | No significant effect |
|  | Increase in pain score % | GTN/mCHI | Sprague-Dawley | F | Fig 7E | Anti-CGRP mAb | No significant effect |
| Christensen et al. 2019 | von Frey (hindpaw) | GTN | C57BL/6J mice | M | Fig 1B | Olcegepant | Antinociception |
|  | von Frey (hindpaw) | GTN | C57BL/6J mice | M | Fig 2B | ALD405 | Antinociception |
|  | von Frey (periorbital) |  | STA | F | Fig 4A | ALD405 | Antinociception |
|  | von Frey (periorbital) |  | STA | F | Fig 4B | Olcegepant | Antinociception |
|  | von Frey (hindpaw) |  | STA | F | Fig 4C | ALD405 | No effect |
|  | von Frey (hindpaw) |  | STA | F | Fig 4D | Olcegepant | No effect |
| Christensen et al. 2020 | von Frey (hindpaw) | GTN | C57BL/6J | M | Fig 2 | Olcegepant (IP route) | Antinociception |
|  | von Frey (hindpaw) | GTN | C57BL/6J | M | Fig 2 | Olcegepant (ICV route) | No effect |
|  | von Frey (hindpaw) | GTN | C57BL/6J | M | Fig 3 | ALD405 (IP route) | Antinociception |
|  | von Frey (hindpaw) | GTN | C57BL/6J | M | Fig 3 | ALD405 (ICV route) | No effect |
| Christensen et al. 2021 | von Frey (cephalic area) | Cilostazol | C57BL/6JBomTac | M/F | Fig 2E | ALD405 | Antinociception |
|  | von Frey (cephalic area) | Cilostazol | C57BL/6JBomTac | M/F | Fig 2F | Olcegepant | Antinociception |
|  | von Frey (cephalic area) | Levcromakalim | C57BL/6JBomTac | M/F | Fig 3B | Olcegepant | Antinociception |
|  | von Frey (cephalic area) | Levcromakalim | C57BL/6JBomTac | M/F | Fig 3C | ALD405 | Antinociception |
|  | von Frey (cephalic area) | Levcromakalim | Ramp1/Ramp1KO | M/F | Fig 3D | Ramp1 KO | Antinociception |
| Kopruszinski et al. 2021 | von Frey (periorbital) | RS/UMB | C57BL/6J mice | F | Fig 3B | Olcegepant  (1 hr after UMB) | No effect |
|  | von Frey (periorbital) | RS/UMB | C57BL/6J mice | F | Fig 3C | Olcegepant  (30 min prior to UMB) | Antinociception |
| Ernstsen et al. 2021 | von Frey (cutaneous) | GTN | C57BL/6NTac | M | Fig 2B | Olcegepant | Antinociception |
| Ernstsen et al. 2022 | von Frey (cephalic) | GTN | RAMP1KO mice | M/F | Fig 2F | Ramp1 KO | Antinociception |
| Greco et al. 2022 | Formalin test (phase I) | Acute NTG | Sprague-Dawley | M | Fig 2 | Olcegepant | No effect |
|  | Formalin test (phase II) | Acute NTG | Sprague-Dawley | M | Fig 2 | Olcegepant | Antinociception |
|  | Formalin test (phase I) | Chronic NTG | Sprague-Dawley | M | Fig 3 | Olcegepant | No effect |
|  | Formalin test (phase II) | Chronic NTG | Sprague-Dawley | M | Fig 3 | Olcegepant | Antinociception |
| Romero-Reyes et al. 2015 | Forepaw face rubbing | CFA | C67BL/6J | F | Fig 1A | MK8825 | Antinociception |
|  | Chin/cheek rubbing | CFA | C67BL/6J | F | Fig 1B | MK8825 | Antinociception |
|  | Hindpaw face scratching | CFA | C67BL/6J | F | Fig 1C | MK8825 | Antinociception |
| Munro et al. 2018 | von Frey (periorbital) | STA | STA rats | F | Fig 6B | Olcegepant | Antinociception |
|  | von Frey (hindpaw) | STA | STA rats | F | Fig 6F | Olcegepant | No effect |
| Guo et al. 2021 | von Frey (acute sensitization) | NTG | CGRPα^EGFPf/+^ mice | F | Fig 1B | CGRPα KO | Blocked NTG-induced hypersensitivity |
|  | von Frey (persistent sensitization) | NTG | CGRPα^EGFPf/+^ mice | F | Fig 1C | CGRPα KO | Blocked NTG-induced hypersensitivity |
| Chou et al. 2022 | von Frey | NTG | C57BL/6J mice | M/F | Fig 4C | CGRP_8-37_ | Antinociception |
| Viero et al. 2022 | von Frey (hindpaw) | USS | C57BL/6J mice | M | Fig 5A | BIBN4096BS | Antinociception |
|  | von Frey (hindpaw) | USS | C57BL/6J mice | F | Fig 5B | BIBN4096BS | Antinociception |
|  | von Frey (periorbital) | USS | C57BL/6J mice | M | Fig 5D | BIBN4096BS | Antinociception |
|  | von Frey (periorbital) | USS | C57BL/6J mice | F | Fig 5E | BIBN4096BS | Antinociception |
|  | Grimacing | USS | C57BL/6J mice | M | Fig 5G | BIBN4096BS | Antinociception |
|  | Grimacing | USS | C57BL/6J mice | F | Fig 5H | BIBN4096BS | Antinociception |
|  | Open field (grooming) | USS | C57BL/6J mice | M | Fig 6A | BIBN4096BS | Decreased anxiety-like behavior |
|  | Open field (grooming) | USS | C57BL/6J mice | F | Fig 6A | BIBN4096BS | Decreased anxiety-like behavior |
|  | Open field (sniffing) | USS | C57BL/6J mice | M | Fig 6B | BIBN4096BS | Reverted exploratory behavior |
|  | Open field (sniffing) | USS | C57BL/6J mice | F | Fig 6B | BIBN4096BS | Reverted exploratory behavior |
|  | Open field (rearing) | USS | C57BL/6J mice | M | Fig 6C | BIBN4096BS | Reverted exploratory behavior |
|  | Open field (rearing) | USS | C57BL/6J mice | F | Fig 6C | BIBN4096BS | Reverted exploratory  Behavior |
|  | Open field (crossings) | USS | C57BL/6J mice | M | Fig 6D | BIBN4096BS | No effect |
|  | Open field (crossings) | USS | C57BL/6J mice | F | Fig 6D | BIBN4096BS | Reverted exploratory behavior |
|  | Open field (peripheral zone) | USS | C57BL/6J mice | M | Fig 6F | BIBN4096BS | Reverted anxiety-like behavior |
|  | Open field (peripheral zone) | USS | C57BL/6J mice | F | Fig 6F | BIBN4096BS | Reverted anxiety-like behavior |
| Wu et al. 2022 | von Frey (periorbital) | NTG | C57BL/6J mice | M | Fig 1B | Olcegepant | Antinociception |
|  | von Frey (hindpaw) | NTG | C57BL/6J mice | M | Fig 1C | Olcegepant | Antinociception |
|  | von Frey (periorbital) | LEV | C57BL/6J mice | M | Fig 5B | Olcegepant | Antinociception |
|  | von Frey (hindpaw) | LEV | C57BL/6J mice | M | Fig 5C | Olcegepant | Antinociception |
| Navratilova et al. 2025 | von Frey (periorbital)  (APTH) | mTBI | ICR mice | F/M | Fig 1  A-B | Olcegepant | Antinociception* |
|  | von Frey (periorbital)  (PPTH – day 14) | mTBI/  BLS | ICR mice | F/M | Fig 1  D-E | Olcegepant | Antinociception* |
|  | von Frey (periorbital)  (PPTH – day 28) | mTBI/  BLS | ICR mice | F/M | Fig 1  G-H | Olcegepant | Antinociception* |
|  | von Frey (hindpaw)  (APTH) | mTBI | ICR mice | F/M | Fig 2  A-B | Olcegepant | Antinociception* |
|  | von Frey (hindpaw)  (PPTH – day 14) | mTBI/  BLS | ICR mice | F/M | Fig 2  D-E | Olcegepant | Antinociception* |
|  | von Frey (hindpaw)  (PPTH – day 28) | mTBI/  BLS | ICR mice | F/M | Fig 2  G-H | Olcegepant | Antinociception* |
|  | Thermal allodynia  (APTH) | mTBI | ICR mice | F/M | Fig 3  A-B | Olcegepant | Antinociception* |
|  | Thermal allodynia  (PPTH – day 14) | mTBI/  BLS | ICR mice | F/M | Fig 3  D-E | Olcegepant | Antinociception* |
|  | Thermal allodynia  (PPTH – day 28) | mTBI/  BLS | ICR mice | F/M | Fig 3  G-H | Olcegepant | Antinociception* |
|  | von Frey  (PPTH – day 14) | mTBI/BLS | ICR mice | F/M | Fig 4  A-B | Olcegepant  (after PPTH) | No effect |
|  | von Frey  (PPTH – day 28) | mTBI/BLS | ICR mice | F/M | Fig 4  D-E | Olcegepant  (after PPTH) | No effect |

**Abbreviations:** APTH = acute post-traumatic headache; CFA = complete Freund’s adjuvant; CCI = controlled cortical impact; GTN = glyceryl trinitrate; ISCap = capsaicin and inflammatory mediators ; LPS = lipopolysaccharide ; mCHI = mild closed head injury; mTBI=mild traumatic brain injury; NTG = nitroglycerin; PPTH = persistent post-traumatic headache; RS = restraint stress; STA = spontaneous trigeminal allodynia; UMB = umbellulone; USS = unpredictable sound stress *Indicates sex-differences in efficacy of CGRP antagonism were observed.
