# Supplementary material for: Sex differences in the effects of calcitonin gene-related peptide signaling on migraine-like behavior in animal models: a narrative review
Source: Front Neurol. 2025 Jul 10;16:1603758. doi: 10.3389/fneur.2025.1603758 (PMC12288688; doi:10.3389/fneur.2025.1603758)
Supplement: Supplementary file 2 [file Table_2.docx]

**
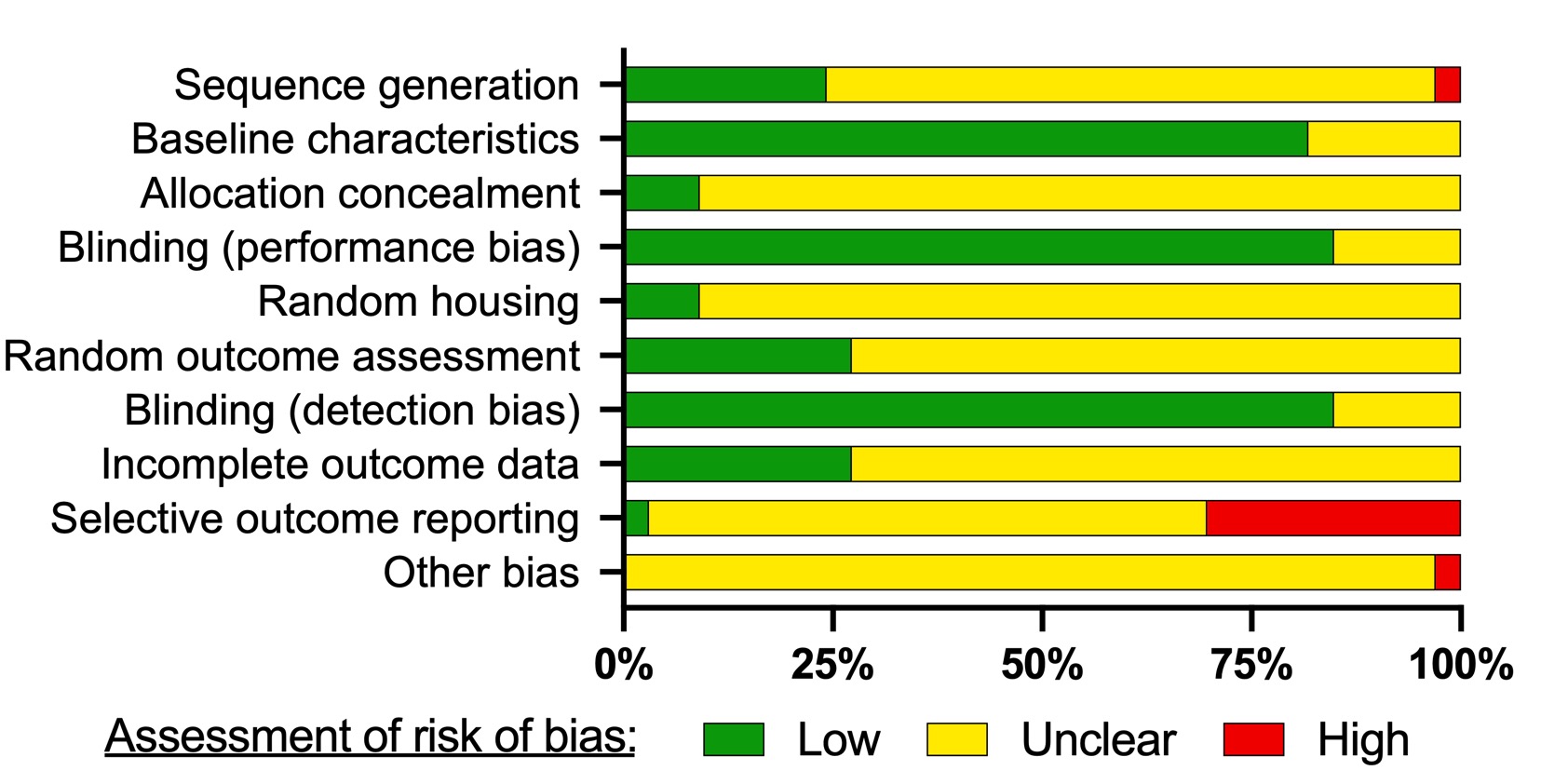
**

**Figure 1: Risk of bias assessment.** The SYRCLE’s risk of bias tool for animal studies was used to assess the risk of bias for each study included in the review. The risk of bias is presented as a percentage of the 35 included studies. Articles were evaluated according to the following domains: 1) sequence generation: was the allocation sequence adequately generated and applied? 2) baseline characteristics: were the groups similar at baseline or were they adjusted for confounders in the analysis? 3) allocation concealment: was the allocation adequately concealed? 4) blinding (performance bias): were the caregivers and/or investigators blinded from knowledge which intervention each animal received during the experiment? 5) random housing: were the animals randomly housed during the experiment? 6) random outcome assessment: were animals selected at random for outcome assessment? 7) blinding (detection bias): was the outcome assessor blinded? 8) incomplete outcome data: were incomplete outcome data adequately addressed? 9) selective outcome reporting: are reports of the study free of selective outcome reporting? 10) other sources of bias: was the study apparently free of other problems that could result in high risk of bias? (e.g. a wide range of n values across groups). Each type of bias classified as low, medium, or high risk of bias.


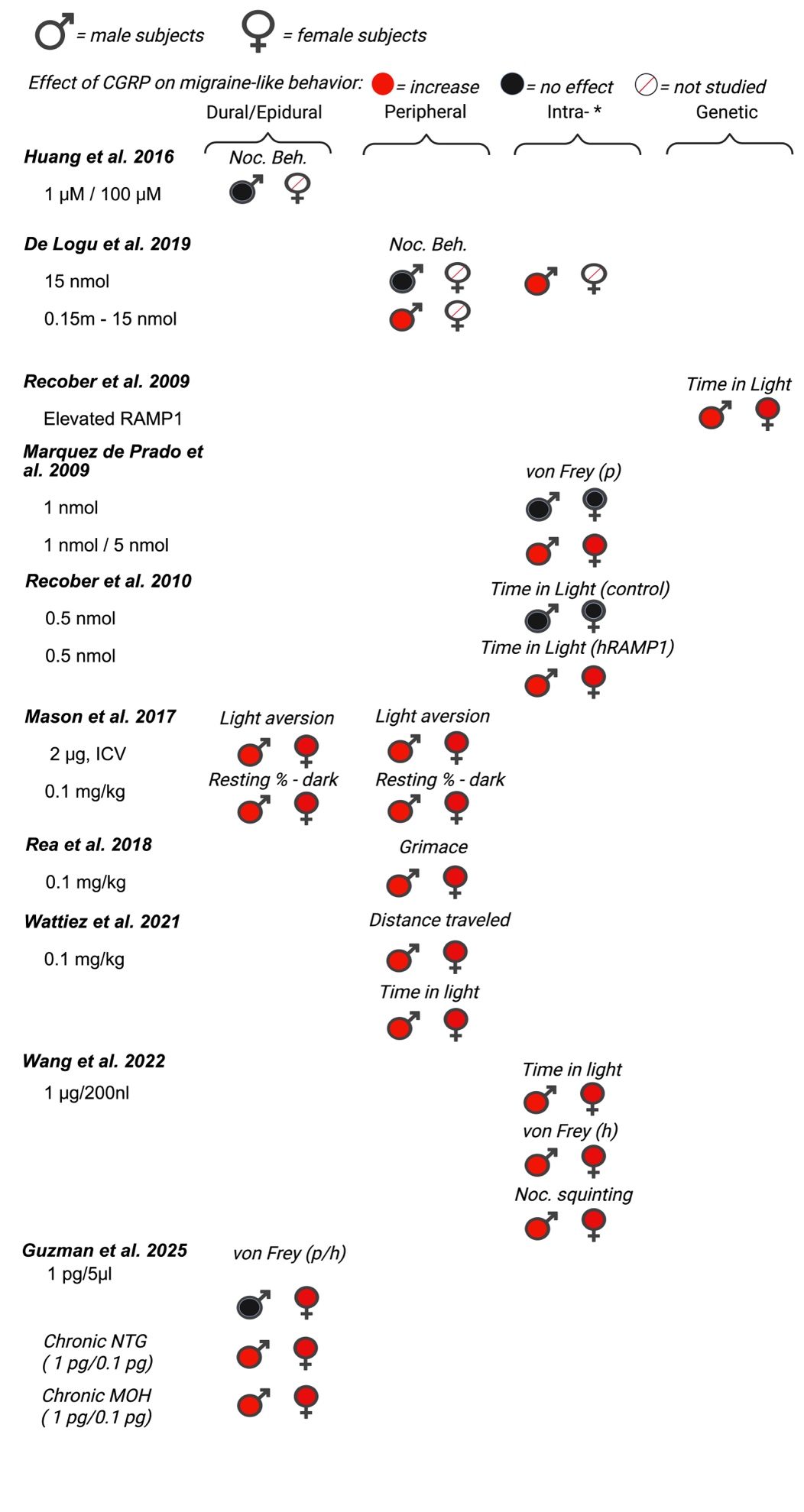


**Figure 2: Summary of sex differences in studies assessing the role of CGRP on migraine-like behavior in mice.** Studies are categorized by route of administration of CGRP (dural/epidural, peripheral, *intra-[intrathecal, intracisternal, intracerebroventricular, intraganglionar], and transgenic mouse model [nestin/hRAMP1]. Black, filled symbols indicate CGRP agonism had no effect on migraine-like behavior. Red, filled symbols indicate CGRP agonism increased migraine-like behavior. A red, diagonal line through the symbol indicates that CGRP agonism was not assessed in that particular sex. (p) = periorbital, (h) = hindpaw


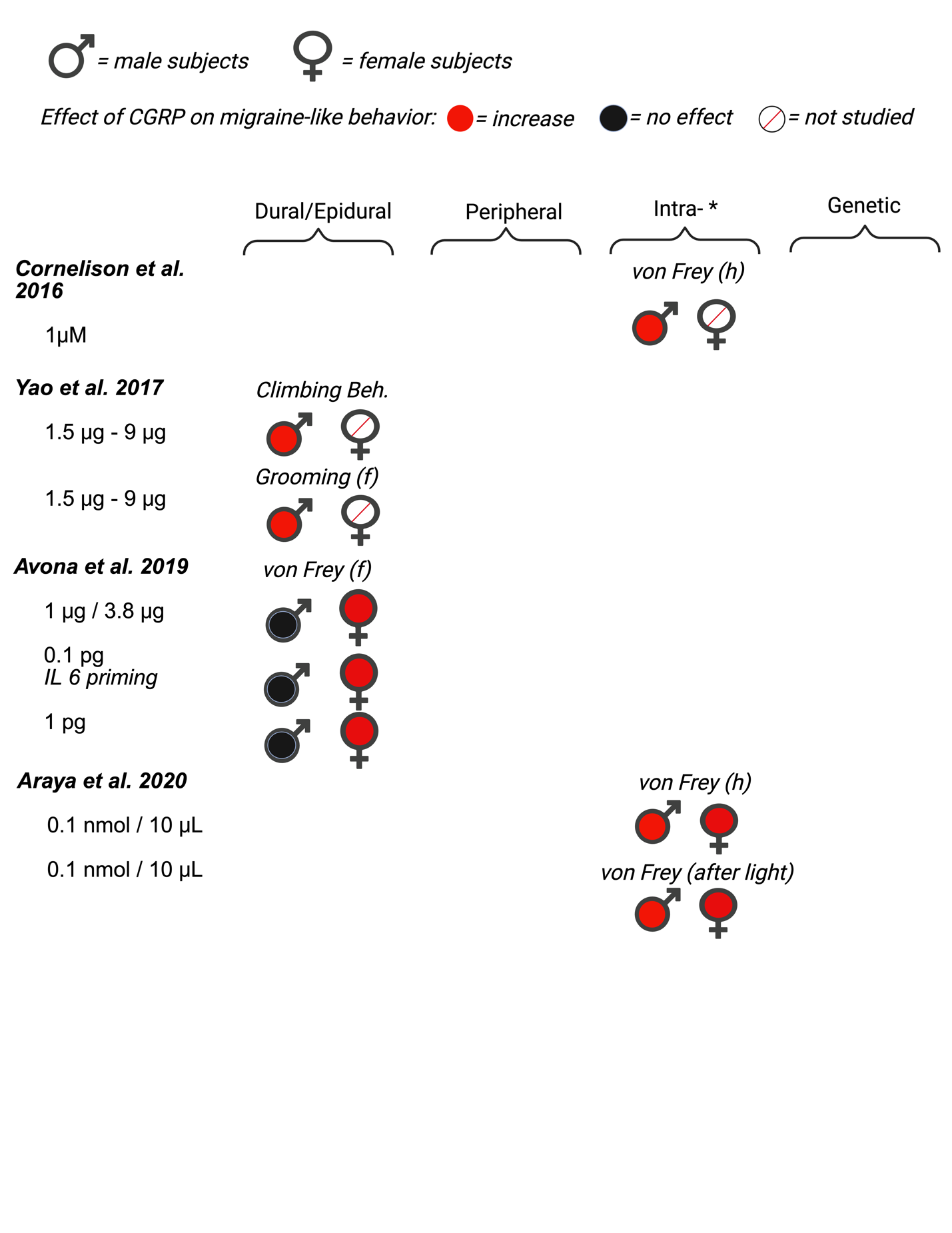


**Figure 3: Summary of sex differences in studies assessing the role of CGRP on migraine-like behavior in rats.** Studies are categorized by route of administration of CGRP (dural/epidural, peripheral, *intra-[intrathecal, intracisternal, intracerebroventricular, intraganglionar], and transgenic mouse model [nestin/hRAMP1]. Black, filled symbols indicate CGRP agonism had no effect on migraine-like behavior. Red, filled symbols indicate CGRP agonism increased migraine-like behavior. A red, diagonal line through the symbol indicates that CGRP agonism was not assessed in that particular sex. (f) = face/facial, (h) = hindpaw


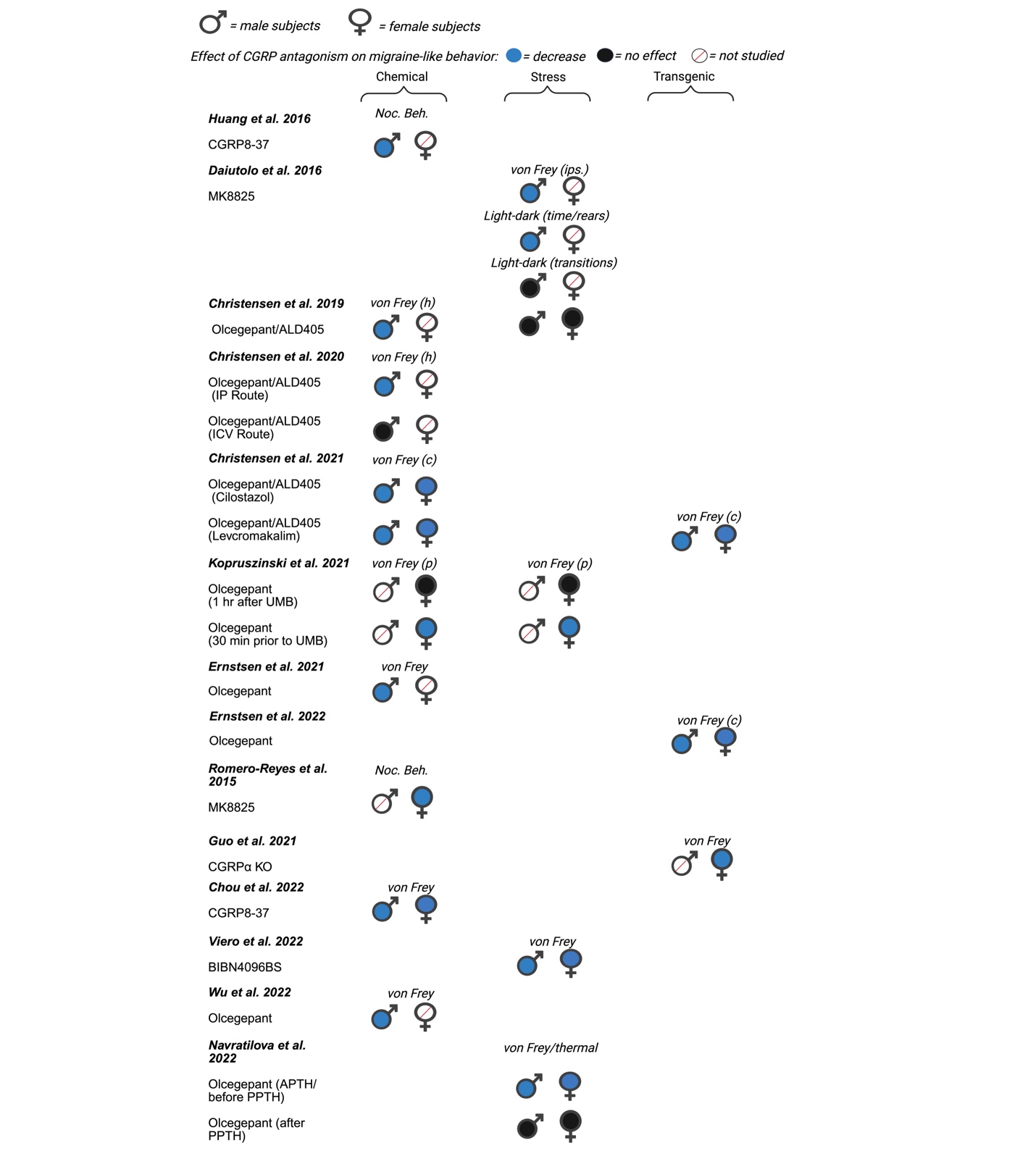


**Figure 4: Summary of sex differences in studies assessing the role of CGRP antagonism on migraine-like behavior in mice.** Studies are categorized by model of migraine/headache (chemical, stress, and transgenic). Black, filled symbols indicate CGRP antagonism had no effect on migraine-like behavior. Blue, filled symbols indicate CGRP antagonism decreased migraine-like behavior. A red, diagonal line through the symbol indicates that CGRP antagonism was not assessed in that particular sex. (ips.) = ipsilateral, (p) = periorbital, (c) = cephalic, (h) = hindpaw


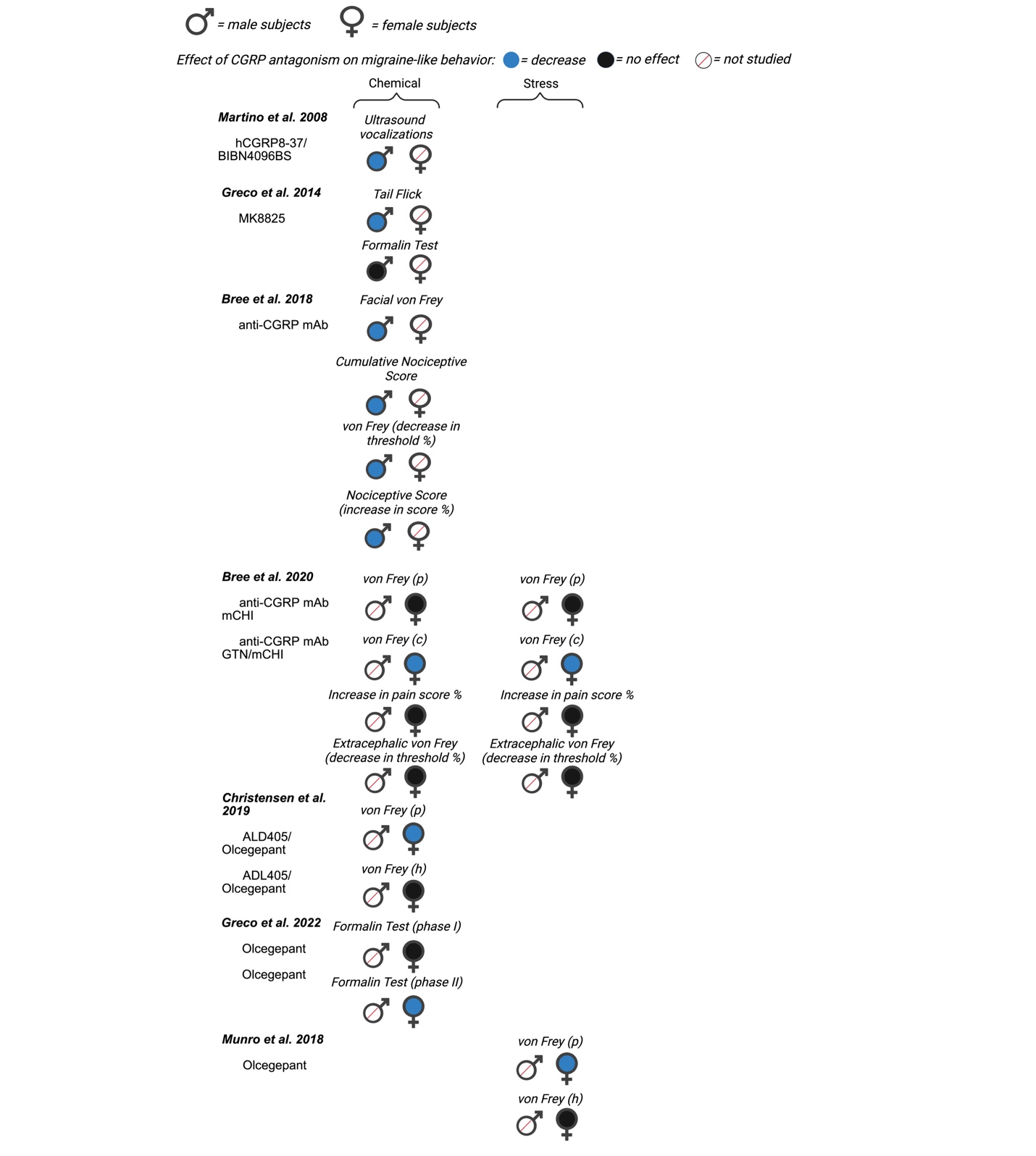


**Figure 5: Summary of sex differences in studies assessing the role of CGRP antagonism on migraine-like behavior in rats.** Studies are categorized by model of migraine/headache (chemical, stress, and transgenic). Black, filled symbols indicate CGRP antagonism had no effect on migraine-like behavior. Blue, filled symbols indicate CGRP antagonism decreased migraine-like behavior. A red, diagonal line through the symbol indicates that CGRP antagonism was not assessed in that particular sex. (p) = periorbital, (c) = cephalic, (h) = hindpaw


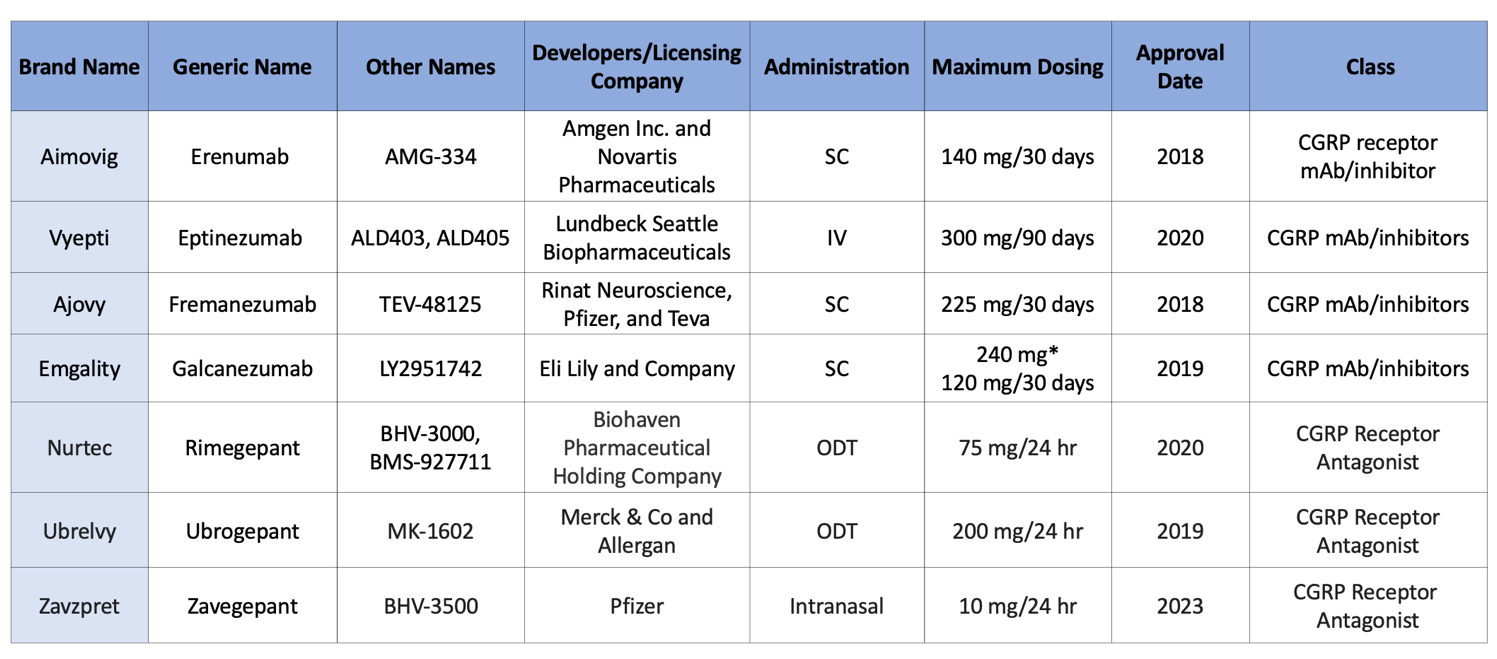


**Table 1:** ***FDA Approved CGRP-Targeting Monoclonal Antibodies and Antagonists for Migraine.*** SC = subcutaneous, IV = intravenous, ODT = orally disintegrating tablet. * The loading dose of emgality is 240 mg SC (2 consecutive 120 mg SC injections) and the maintenance dose is 120 mg per month. Dosing values are for human clinical use.
